# Early Neolithic genomes suggest a local origin for millet farmers in East Asia

**DOI:** 10.1126/sciadv.aef9182

**Published:** 2026-10-16

**Authors:** Tingyu Yang, Xiaowen Jia, Jincheng Wang, Tao Wang, Tianxing Cui, Chunmei Li, Jianing He, Tianming Zhang, Zhanhu Zhao, Jiaojiao Hao, Yingjie Cui, Jincheng Yu, Zhonghua Zhang, Jingning Guo, Qihuang Yang, Shi Yan, Xiaohong Wu, Chaohong Zhao, Yuhong Pang, Yanyi Huang

**Affiliations:** ^1^ Biomedical Pioneering Innovation Center, Peking University, Beijing 100871, China.; ^2^ College of Chemistry and Molecular Engineering, Beijing National Laboratory for Molecular Sciences, Peking University, Beijing 100871, China.; ^3^ Beijing Institute of Genomics, China National Center for Bioinformation, Chinese Academy of Sciences, Beijing 100101, China.; ^4^ School of Archaeology and Museology, Peking University, Beijing 100871, China.; ^5^ School of History, Capital Normal University, Beijing 100048, China.; ^6^ School of Archaeology and Cultural Heritage, Zhengzhou University, Zhengzhou 450001, China.; ^7^ Bio-X Center for Interdisciplinary Innovation, Yunnan University, Kunming 650500, China.; ^8^ Peking-Tsinghua Center for Life Sciences, Peking University, Beijing 100871, China.; ^9^ Academy for Advanced Interdisciplinary Studies, Peking University, Beijing 100871, China.; ^10^ Hebei Provincial Institute of Cultural and Archaeology, Shijiazhuang 050031, China.; ^11^ Yulin Municipal Cultural Relics Archaeology and Exploration Work Team, Yulin 719000, China.; ^12^ Beijing Institute of Archaeology, Beijing 100078, China.; ^13^ Capital Museum, Beijing 100045, China.; ^14^ Shaanxi Academy of Archaeology, Xi’an 710109, China.; ^15^ School of Ethnology and Sociology, Minzu University of China, Beijing 100081, China.; ^16^ Key Laboratory of Archaeological Science (Peking University), Ministry of Education, Beijing 100871, China.; ^17^ Institute of Chemical Biology, Shenzhen Bay Laboratory, Guangdong 518132, China.

## Abstract

Millet cultivation in northern China served as a critical catalyst for Neolithic sedentism and East Asian civilization, yet the demographic origins of the inaugural millet farmers remain contentious. We analyze genome-wide data from seven individuals from the Donghulin (DHL), Dengcaogouliang, and Sitai sites, spanning 11,000 to 7300 years ago, and identify the DHL lineage as the predominant genetic structure in inland northern East Asia following the Last Glacial Maximum. Our results demonstrate profound genetic continuity from the Early Holocene through the intensification of millet agriculture, lending robust support to a model of local development rather than demic diffusion from external territories. Furthermore, we identify the earliest occurrence of the hypoxia-adaptive *EGLN1* variant in a 9200-year-old DHL individual, suggesting that the physiological adaptations currently prevalent in Tibeto-Burman populations have deep Neolithic roots. Collectively, these findings necessitate a reevaluation of the early demographic and adaptive landscape of East Asia.

## INTRODUCTION

Millet cultivation originated in northern China, an early center of crop domestication (*1*–*3*). The millet-based agricultural system has brought sedentism and food production, profoundly influenced the development of ancient societies in East Asia (*4*, *5*), and disseminated across various Eurasian societies (*6*–*8*). Evidence from phytoliths (*9*), starch grains (*10*), stable isotopes (*11*), and carbonized millets with indirect dating (*12*) has extended the archeological record of millet exploitation to ∼11,000 years before the present (yr B.P.). After ∼8000 yr B.P., millet agriculture expanded across northern China, including the Yellow River Basin, the West Liao River Basin, the Inner Mongolian Plateau, the Loess Plateau, and even into regions less favorable for millet cultivation, such as the Tibetan Plateau (*13*–*15*).

The expansion of millet agriculture was postulated to have been driven by human migration (*16*). Supporting this, paleogenetic studies have established distinct genetic profiles for Middle and Late Neolithic farming populations in China and documented several dispersal events associated with millet agriculture, including the spread of Upper Yellow River farmer–related ancestry onto the Tibetan Plateau during the Late Neolithic (*17*–*19*), the increase of Yellow River farmer–related ancestry in West Liao River populations with the intensification of millet (*20*), and southward expansion of Yellow River farmers toward the Yangtze River region (*21*, *22*). These studies demonstrate the importance of population movement in the expansion of millet agriculture during the Middle and Late Neolithic period. However, the demographic history underlying its origins and earlier spread in northern China remains unclear, largely because the relevant ancient genomes are currently scarce. To date, only a limited number of ancient genomes of Late Paleolithic to Early Neolithic foragers (*23*, *24*) and Early Neolithic millet farmers (*25*, *26*) in northern China have been reported. As a result, it remains unresolved whether early use and domestication of millet was driven by indigenous hunter-gatherers or by incoming farming populations (*27*).

To address this gap, we obtained genome-wide data from skeletal remains of seven individuals recovered from key archeological sites in northern China (Fig. 1A), encompassing a temporal transect from the beginning of the Holocene. This includes the Donghulin (DHL) site, which belongs to the Paleolithic-to-Neolithic transition period (*n* = 3, spanning approximately 11,000 to 9000 years ago, located in Beijing, China), and two early millet farming sites: the Dengcaogouliang (DCGL) site (*n* = 2) and the Sitai (ST) site (*n* = 2), both located in Zhangjiakou (ZJK), Hebei Province, China, and dating from 7900 to 7300 years ago. We also report carbon and nitrogen isotopic data, including 18 individuals from the DHL, DCGL, and ST sites, as well as one individual from the Yumin site for which ancient DNA data had been previously published (*23*). By comparing the genetic profiles of DHL individuals with those of later millet-associated populations from the nearby ZJK region, we directly test whether early millet cultivation and its later intensification occurred within a local genetic background or were associated with population replacement.

**Fig. 1.**
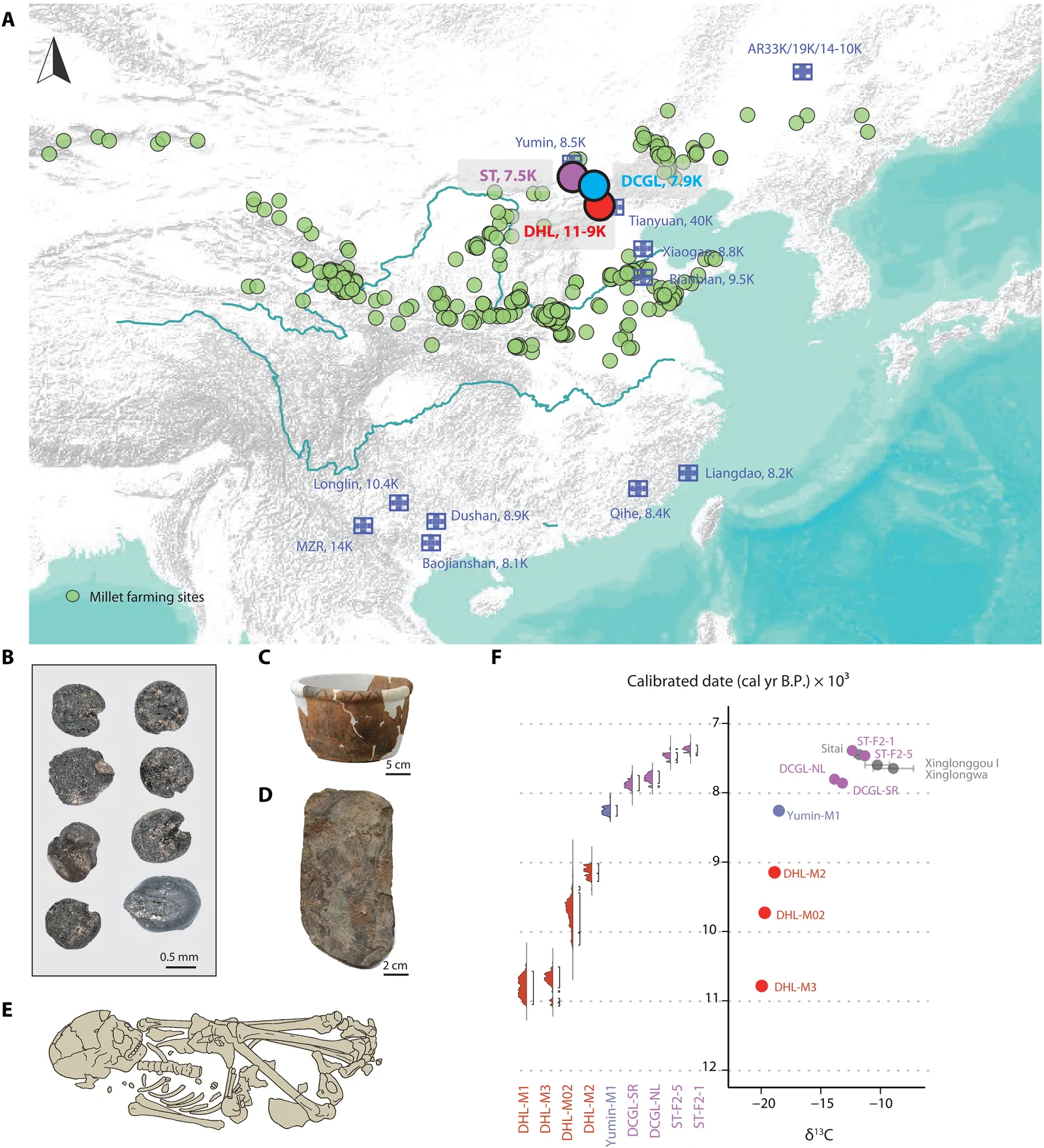
Geographical locations and archeological information of early millet cultivation–related populations. (**A**) Geographical locations of analyzed samples, with previously published Late Pleistocene and Early Holocene individuals. The spatial distribution of millet farming sites is indicated by green dots. The base map was obtained from the Word Terrain Base domain map dataset (https://www.arcgis.com/home/item.html?id=c61ad8ab017d49e1a82f580ee1298931) and created with ArcGIS Pro v3.0. Sources: Esri, US Geological Survey (USGS), and National Oceanic and Atmospheric Administration. (**B**) A total of 14 carbonized millet grains was identified in the flotation samples from the DHL site, 8 of which were photographed under a stereomicroscope. These millet grains exhibit a spherical or near-spherical morphology, with slender embryos, and feature characteristic furrows formed during the carbonization process. (**C** to **E**) Archeological remains excavated from the DHL site. (C) Restored pottery. (D) Stone knife. (E) Human skeleton of individual M2 in a supine, flexed burial posture. Drawn using Adobe Illustrator based on the actual excavation photograph of the tomb. (**F**) The left panel shows the Bayesian chronological model for the calibrated accelerator mass spectrometry (AMS) ^14^C dates of DHL, DCGL, ST, and Yumin individuals, displaying 68.2 and 95.4% confidence intervals along with posterior probability distributions. The right panel displays the radiocarbon dates and corresponding δ^13^C values for these individuals, with density plots used to illustrate the radiocarbon dates and δ^13^C ranges of individuals from early millet farming sites (ST, Xinglonggou I, and Xinglongwa) (photo credit: C.Z.; Peking University).

Our analyses reveal that the DHL individuals represent a predominant genetic structure in inland northern East Asia (nEA) after the Last Glacial Maximum (LGM), showing genetic affinities with hunter-gatherers from the Amur River Basin. We further reveal a close genetic continuity between DHL and the early farming groups DCGL and ST, suggesting that they belong to the same genetic branch that initiated millet agriculture. Our findings fill a critical gap in understanding the demographic structure of early millet cultivators and their impact on later farmers.

## RESULTS

### Background and sampling at DHL, DCGL, and ST sites

During the transition from the Paleolithic to the Neolithic era in East Asia, along with a notable warming of the climate, early modern humans began to implement and disseminate stone grinding technology, innovate pottery, and develop rudimentary forms of agriculture and animal husbandry. Consequently, there was a marked shift in subsistence strategies from hunting and gathering to sedentary agriculture (*28*). The DHL site (39.99°N to 115.75°E) is located on the northwestern fringe of the North China Plain (Fig. 1A), dating back to approximately 11,000 years ago following the end of the Last Ice Age (*10*, *12*, *29*). Archeological excavations have recovered the earliest known physical remains of carbonized millet seeds (Fig. 1B), along with numerous fire pits, various grain-processing tools including stone grinding implements (Fig. 1, C and D, and fig. S1), and the remnants of human-built shelters. The morphological characteristics of millet indicate that it had been domesticated or was in the process of domestication. From this site, at least seven human remains were recovered; however, only four individuals were available for ancient DNA analysis, as the other three remains are currently missing and could not be sampled. These four individuals included two relatively complete skeletons (M1 and M2) (Fig. 1E) and two partial skeletons (M3 and M02). The skeletal elements sampled for DNA extraction are summarized in data S1. Direct radiocarbon dating showed that these individuals ranged from 11,000 to 9200 yr B.P. (data S1). We used shotgun sequencing and targeted-loci enrichment to recover ancient DNA of these four individuals. For individual M02, only mitochondrial DNA (mtDNA) was successfully retrieved, enabling haplotype analysis. Ultimately, sufficient sequencing depth on the 1240k panel (*30*, *31*) was obtained for M1 (0.80× coverage), M2 (2.24× coverage), and M3 (0.86× coverage) (data S2 and S3), enabling robust downstream genetic analyses.

The ZJK region is geographically proximal to the DHL site and includes two Early Neolithic sites: ST (41.29°N to 114.23°E) and DCGL (40.87°N to 114.93°E) (Fig. 1A). Archeological evidence suggests that millet cultivation was adopted in this area as early as around 7500 yr B.P. (*32*). We extracted ancient DNA from the petrous bones of four individuals dating to between 7900 and 7300 calibrated years before the present (cal yr B.P.). Subsequent shotgun genome sequencing yielded four high-quality datasets, including two individuals from ST (3.20× and 3.71×; mean coverage across 1240k sites) and two from DCGL (1.01× and 2.09×; mean coverage across 1240k sites) (data S2 and S3). The authenticity of all datasets from the three sites was confirmed via the characteristic postmortem chemical damage patterns of ancient DNA (fig. S2) and a minimal level of modern human DNA contamination (with mitochondrial estimates for all eight individuals and nuclear estimates for five males, all below 3%). Notably, genomic data from the ST site were recently reported in two independent studies (*25*, *26*). To maximize the statistical power of our population genetic analyses while avoiding data redundancy due to overlapping individuals between these two studies, we integrated our ST data with the recently reported Sitai7.6k dataset (*26*), the larger of the two published ST datasets, for further analysis.

In addition, we obtained stable carbon (δ^13^C) and nitrogen (δ^15^N) isotope data from 19 individuals associated with early millet cultivation at the DHL, ST, and DCGL sites, as well as the Yumin site, an early Neolithic settlement geographically proximate to our study area (data S1 and S4). Combined with previously published data (data S4), these results show a trajectory of increasing δ^13^C values from 11,000 to 7000 yr B.P. (Fig. 1F). In the earlier period, DHL individuals exhibit δ^13^C values ranging from −19.95 to −18.88 per mil (‰), characterizing a subsistence strategy primarily based on C3 resources with moderate C4 consumption. In the subsequent period, while the Yumin individual retains a δ^13^C value similar to that of DHL, ST and DCGL individuals (*n* = 15) exhibit markedly higher δ^13^C values (−13.85 to −11.25‰), reflecting gradually intensified consumption of C4 plants (most likely millet), marking a notable shift in dietary structure.

### Genetic coherence and phylogenetic position of the DHL lineage

We first focused on the earliest three individuals: DHL-M1 (∼11,000 yr B.P.), DHL-M3 (∼10,700 yr B.P.), and DHL-M2 (∼9200 yr B.P.). Despite spanning nearly two millennia, these individuals exhibit a coherent genetic structure. Principal components analysis (PCA) reveals strong genetic affinities among them (Fig. 2A and fig. S3). This is formally confirmed by *f*_4_-statistics using 23 previously published ancient nEA groups as test populations (detailed population information and corresponding references are provided in data S5 and S10). For all test populations used, the form *f*_4_(Mbuti, DHL_x; Test, DHL_y) (where “Test” denotes the specific test population and DHL_x and DHL_y represent different DHL individuals) consistently yielded results greater than zero (0.09 ≤ *Z* ≤ 7.92), with ∼80% of cases showing *Z* > 2.5. Complementarily, the configuration *f*_4_(Mbuti, Test; DHL_x, DHL_y) yielded near-zero values with −1.6 ≤ *Z* ≤ 2.29 (data S5), demonstrating that none of the DHL individuals shared excess genetic affinity with the previously published nEA groups. Furthermore, qpWave modeling demonstrates that the genetic variation among DHL individuals is consistent with descent from a single ancestral stream (*P* > 0.05; fig. S4). On the basis of these results, we merged the three DHL individuals into a single group for subsequent analyses.

**Fig. 2.**
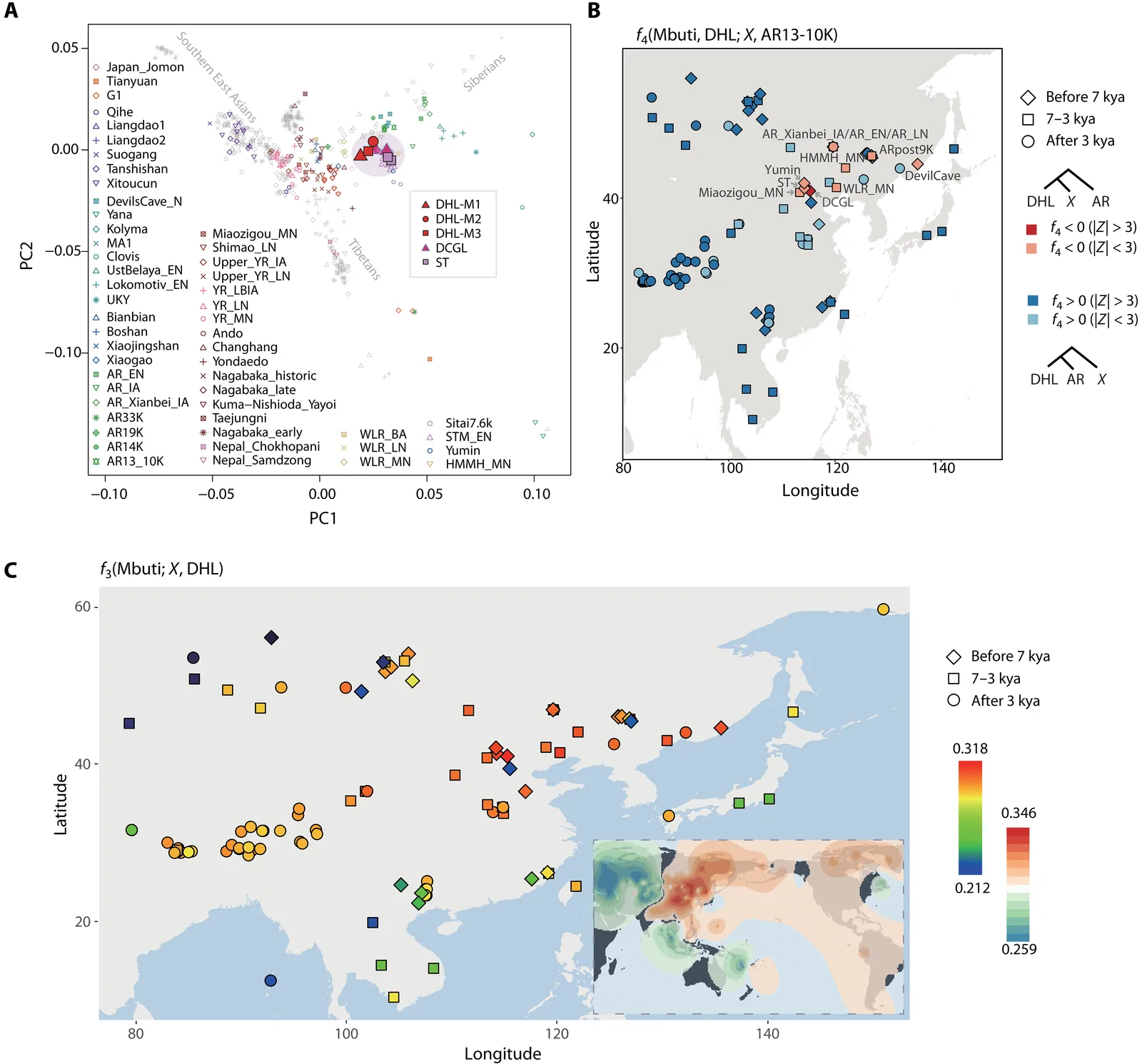
Genetic characteristics of DHL population. (**A**) PCA with ancient Asians (colored symbols, including samples from previous studies) projected onto the genetic diversity of present-day East Asians (gray symbols). Present-day East Asians in the background form three trajectories directed toward Southern East Asia, Siberia, and Tibetan Plateau, respectively. ST, STM_EN, and Sitai7.6k refer to samples from the same archeological site. (**B**) *f*_4_(Mbuti, DHL; Test, AR13-10K) tests the genetic affinities of DHL compared to the AR13-10K population, with different symbols distinguishing the age of test samples. Maps were generated in R using the ggplot2 package, with coastlines derived from the public-domain Natural Earth dataset (www.naturalearthdata.com). (**C**) *f*_3_(Mbuti; *X*, DHL) for genetic drift analysis where *X* represents ancient populations in Eurasia. The *f*_3_ values range from 0.212 to 0.318. A higher value (red) indicates higher shared genetic drift with DHL. The small inset in the bottom right corner shows the spatial distribution of *f*_3_ values for worldwide populations, fitted using the kriging method. The background basemap is the same as in (B).

We then investigated the phylogenetic relationship of DHL within the broader nEA genetic landscape. As shown in PCA results, when compared to earlier or contemporaneous ancient individuals from Siberia and nEA, DHL shows closer genetic clustering with Amur River (AR) populations (*24*) (AR19K, AR14K, AR13-10K) than with Ancient Paleo-Siberians [APS; represented by UKY (*33*) and Kolyma1 (*34*)] (Fig. 2A and fig. S3). Furthermore, compared to AR19K, the earliest known nEA individual after the LGM, DHL exhibits significantly stronger affinity to later nEA populations as indicated by the negative *z*-scores of the test *f*_4_(Mbuti, DHL; Test, AR19K) (−6.81 < *Z* < −1.41) and positive *z*-scores of *f*_4_(Mbuti, Test; AR19K, DHL) (1.22 < *Z* < 7.7). Moreover, the result of symmetry tests in the form of *f*_4_(Mbuti, AR19K; Test, DHL) yielded near-zero results, further supporting AR19K as a basal outgroup (data S5).

In contrast, compared to a broad panel of East Asian populations, DHL shows genetic affinities with post-19K AR hunter-gatherers, such as AR14K and AR13-10K. Statistics of the form *f*_4_(Mbuti, DHL; Test, AR13-10K) indicate that Siberian and southern East Asian populations are generally symmetrically related to DHL and AR13-10K (Fig. 2B). Notably, negative *f*_4_ values were exclusively observed in populations distributed along the geographic corridor extending from the Amur River Basin to northern inland regions, suggesting that the DHL individuals and these populations belong to the same genetic branch, sharing a common ancestral gene pool despite disparities in their subsistence strategies. The outgroup-*f*_3_ statistics [*f*_3_(Mbuti; DHL, Test] provide further corroboration, indicating a shared genetic component that extended across the Amur River Basin, West Liao River, and DHL region during the Early-to-Middle Neolithic (Fig. 2C). In addition, mitochondrial phylogenetic analysis confirms this deep connection, revealing a close maternal relationship between the DHL and AR populations dating back over 11,000 years (fig. S5), despite their Y-chromosome haplogroups belonging to distinct paternal lineages, specifically haplogroup N1a1a-F2209 for DHL-M2/DHL-M3 (data S3) and haplogroup C for the AR population (*24*).

### Long-term genetic continuity and the local origin of millet farmers

We next investigated the genetic relationship between DHL, the Early Holocene agriculture-associated group, and DCGL and ST, the later millet-farming populations from the ZJK region. In PCA, DCGL and ST tightly cluster with the DHL individuals and show close genetic affinity to the Yumin (∼8400 yr B.P.) (*23*) and HMMH_MN (∼5600 yr B.P.) (Fig. 2A and fig. S3) (*20*). This clustering is statistically supported by outgroup-*f*_3_, *f*_4_ statistics and qpWave modeling (Fig. 3, A to C, and figs. S4 and S6), which reveal strong genetic homogeneity across the DHL, DCGL, ST, Yumin, and HMMH_MN despite their spatiotemporal divergence. Moreover, comparative analyses further show that these groups are broadly symmetric relative to diverse nEA populations from the Amur River Basin (AR14K and AR13-10K), Yellow River Basin (Shimao_LN, Miaozigou_MN, Upper_YR_LN, and YR_MN), and coastal Shandong region (Boshan, Bianbian, Xiaogao, and Xiaojingshan), i.e., *f*_4_(Mbuti, DHL/DCGL/ST/Yumin/HMMH_MN; nEA1, nEA2) ∼ 0 (data S5). These findings characterize early millet-associated populations as a distinct genetic substructure within the nEA ancestry, supporting a model of local genetic origin. This implies that the course of millet agriculture development from the initial cultivation and domestication (DHL) (*12*) to an established system (DCGL and ST) occurred within a shared indigenous inland gene pool dating back to at least 11,000 years.

**Fig. 3.**
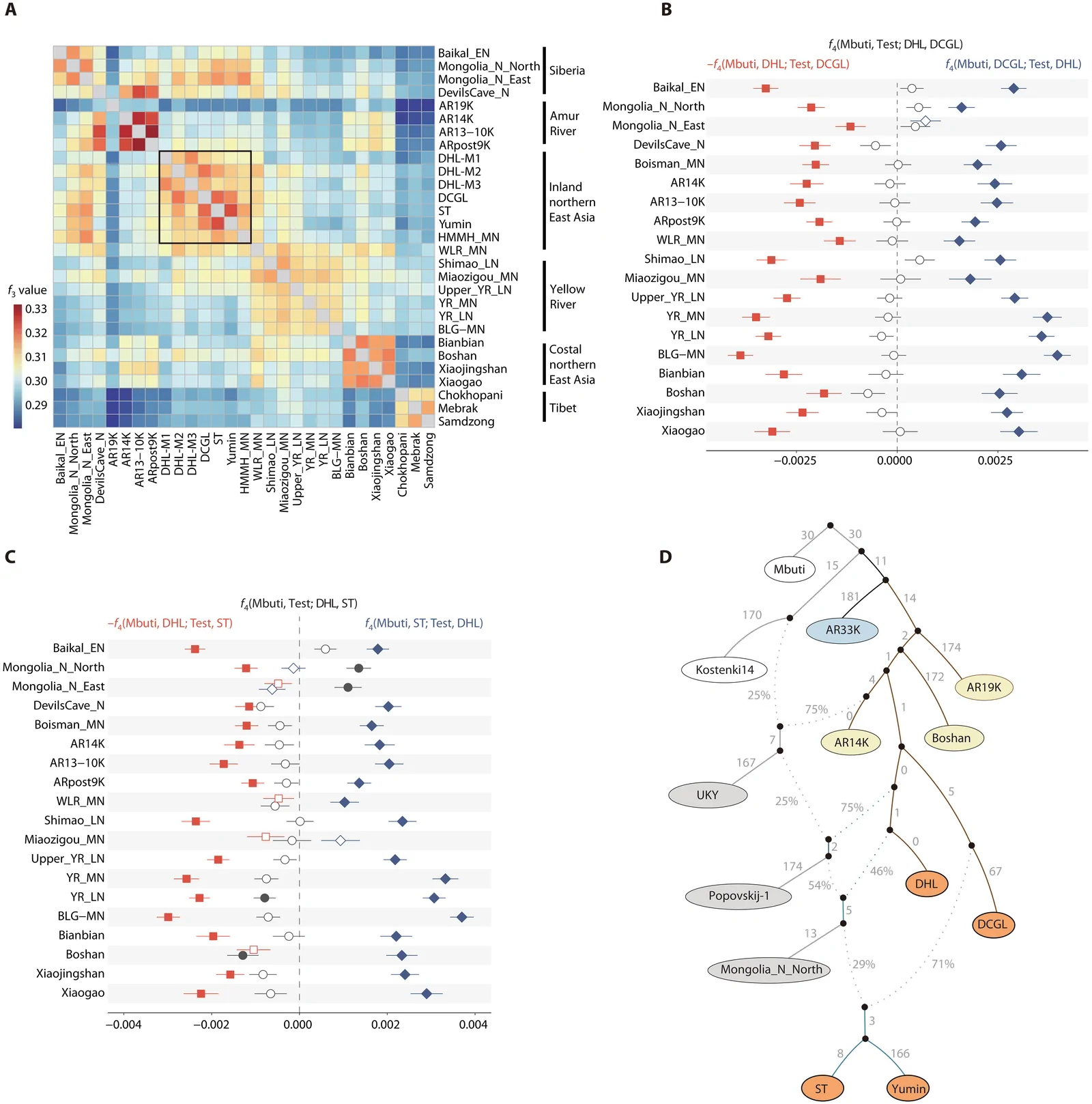
Genetic structure of DHL, DCGL, and ST populations. (**A**) Pairwise outgroup-*f*_3_ analysis using *f*_3_(Mbuti; *X*, *Y*) where *X* and *Y* are ancient nEAs. Red indicates high genetic similarity between pairs, while blue indicates low genetic similarity. (**B**) Genetic affinity tests between DHL and DCGL. (**C**) Genetic affinity tests between DHL and ST. The Test label represents populations from Siberia, the Amur River Basin, the West Liao River Basin, the Yellow River Basin, and coastal Shandong. The dashed line marks *f*_4_ = 0. Solid symbols indicate significant *z*-scores (|*Z*| > 3), and error bars denote one SE. (**D**) Admixture graph modeling inferred with qpGraph. Dashed lines denote admixture events, and orange colored boxes represent Early-to-Middle Neolithic millet-farming populations. The worst residual *z*-score is 2.79.

Within this substructure cluster, to rigorously resolve the internal topological relationships among these five groups (DHL, DCGL, ST, Yumin, and HMMH_MN), we comprehensively evaluated diverse *f*_4_-statistic forms. These included comparisons with external test populations (Fig. 3, B and C, fig. S6, and data S5), alongside internal triplet tests of the form *f*_4_(Mbuti, group1; group2, group3) (fig. S7 and data S5). Our analysis initially highlights that DCGL/ST/Yumin/HMMH_MN all show high genetic affinity to the DHL lineage, i.e., *f*_4_(Mbuti, DCGL/ST/Yumin/HMMH_MN; Test, DHL) > 0 and *f*_4_(Mbuti, DHL; Test, DCGL/ST/Yumin/HMMH_MN) > 0 (data S5), indicating that DHL represents the earliest local ancestral population sampled in this region. On the basis of this foundation, we next observed a strong genetic continuity specifically between DHL and the later DCGL. This temporal stability is externally supported by symmetry against diverse test populations in *f*_4_(Mbuti, Test; DHL, DCGL) ∼ 0 (−1.82 < *Z* < 1.61), coupled with *f*_4_(Mbuti, DHL; Test, DCGL) > 0 (3.14 < *Z* < 12.4) and *f*_4_(Mbuti, DCGL; Test, DHL) > 0 (1.92 < *Z* < 11.9). Internal triplet tests further support this affinity, as DHL shares more alleles with DCGL than with ST, Yumin, or HMMH_MN by *f*_4_(Mbuti, DHL; ST/Yumin/HMMH_MN, DCGL) > 0 (2.29 < *Z* < 2.9) (Fig. 3B and data S5). Together, these consistent statistics demonstrate that DCGL farmers largely retained ancestry associated with DHL without significant external admixture, marking a local genetic lineage stable for more than 3000 years.

Slightly different from this local continuum, ST and Yumin form a highly cohesive clade, with some admixture related to a northern component. First, they exhibit genetic symmetry relative to test populations and internal groups [*f*_4_(Mbuti, Test/DHL/DCGL/HMMH_MN; Yumin, ST) ∼ 0 (−1.14 < *Z* < 2.28)], while both *f*_4_(Mbuti, ST; Test, Yumin) and *f*_4_(Mbuti, Yumin; Test, ST) are significantly positive (4.81 < *Z* < 16.79 and 4.73 < *Z* < 18.78, respectively) (fig. S6 and data S5), indicating that they are sister groups within this clade. Second, *f*_4_-statistics for ST indicate extra affinity with ancient Mongolian and Baikal populations relative to DHL, as shown by positive *f*_4_(Mbuti, Mongolia_N_North/Baikal_EN; DHL, ST) (*Z* = 4.808 and 2.361) (Fig. 3C, fig. S6, and data S5). We then evaluated this dual-ancestry origin involving various nEA and Baikal- and Mongolia-related ancestries using a rotating two-way qpAdm framework (data S6). Crucially, the statistics of all models were rejected when DHL is placed in the outgroup, demonstrating that the DHL-represented ancestry is an indispensable local source for modeling ST and Yumin. In the best-fitting models, ST and Yumin derive 70 to 80% of their ancestry from this local source, admixed with 20 to 30% Baikal- and Mongolia-related ancestry. To pinpoint the exact local source contributing to ST and Yumin, we compared their affinities with DHL and DCGL, respectively. The result shows that ST and Yumin share significantly more derived alleles with the contemporaneous DCGL than with the older DHL, as indicated by *f*_4_(Mbuti, ST/Yumin; DHL, DCGL) > 0 (*Z* = 4.613 and 3.014). DCGL and ST males belong to the same Y-chromosome branch (N1a2a-M178), further supporting the genetic evidence that ST is more closely related to DCGL than to DHL. In the corresponding comparison, *f*_4_(Mbuti, DCGL; ST/Yumin, DHL) is consistent with zero (*Z* = −1.659 and −0.884), likely because the DCGL-related affinity in ST and Yumin is partially counterbalanced by their additional Baikal- and Mongolia-related ancestry.

Last, we investigated the phylogenetic position of the HMMH_MN group relative to these two divergent demographic trajectories (the local continuum versus the admixed ST-Yumin clade). Cross-branch *f*_4_-statistic comparisons place HMMH_MN close to the admixed ST-Yumin branch, as it shares significantly more derived alleles with ST/Yumin than with DHL/DCGL or test populations [*f*_4_(Mbuti, HMMH_MN; DHL/DCGL/Test, ST/Yumin) > 0]. Internally, HMMH_MN is symmetric to ST and Yumin [*f*_4_(Mbuti, HMMH_MN; Yumin, ST) ∼ 0; *Z* = 0.046], a pattern that may be compatible with an early-diverging or parallel relationship to the ST-Yumin clade, although this inference should be interpreted with caution because HMMH_MN is currently represented by only one individual.

Expanding beyond the ZJK region, our analyses reveal that the DHL lineage also exhibits extra affinity to ancient hunter-gatherers further north. Early Neolithic Baikal and Mongolian populations show an overall affinity to nEA ancestry compared to APS-related ancestry, as reflected by positive *f*_4_(Mbuti, DHL/AR/YR/Shandong; UKY, Baikal_EN/Mongolia_N) (4.28 < *Z* < 12.39) (data S5). Direct comparisons among different nEA groups further show that Baikal_EN and Mongolia_N share more derived alleles with the DHL lineage than with Yellow River and Shandong populations, as shown by positive *f*_4_(Mbuti, Baikal_EN/Mongolia_N; YR/Shandong, DHL) (2.09 < *Z* < 8.27) (data S5). Comparisons involving AR-related groups are also positive but do not reach statistical significance, indicating that the current *f*_4_ tests do not determine whether Baikal_EN/Mongolia_N has a stronger affinity to DHL or AR-related lineages. We further tested admixture models for Baikal and Mongolian groups using qpAdm with source rotation. Baikal_EN, Russia_AngaraRiver_EN.SG, and Russia_LenaRiver_EN.SG could be robustly modeled as mixtures of APS-related ancestry and ancestry represented by either DHL or DCGL. For Mongolia_N_North, the best-supported model combined APS-related and DHL-related ancestry, although models combining Baikal-related ancestry with DHL, DCGL, or DevilsCave_N also yielded stable fits (data S7).

Collectively, these findings illuminate a dynamic demographic history across nEA. In an earlier period, DHL ancestry contributed to the formation of Baikal and Mongolian hunter-gatherer populations. Subsequently, Baikal- and Mongolia-related ancestry contributed to the formation of ST and Yumin. These dynamic population interactions are represented by a plausible qpGraph model (Fig. 3D), although alternative topologies yield comparable fits (fig. S8). This model links the local DHL-DCGL continuum, Baikal and Mongolian hunter-gatherers, and the admixed ST-Yumin clade, highlighting a complex and enduring genetic corridor between northern China and the Baikal and Mongolian regions.

### A DHL-related ancestry component in later East Asian populations

On the basis of the genetic affinity between DHL and Middle Neolithic populations from the West Liao River Basin (WLR_MN) (*20*) and nearby regions, including Leigongshan and Zhengjiagou (*26*), as indicated by positive *f*_4_ values of the form *f*_4_(Mbuti, DHL; Test, WLR_MN/Leigongshan/Zhengjiagou) (data S5), we further evaluated this relationship using qpAdm (data S8 and fig. S9). In these models, plausible northern source proxies were rotated between the qpAdm left and right sets, including DHL-related northern inland sources (DHL, Yumin, DCGL, and ST), a northern coastal source (Bianbian), Mongolia- and Baikal-related sources (Baikal_EN and Mongolia_N_North), and ancient Amur River Basin– and Northeast Asian–related sources (AR14K and DevilsCave_N). For WLR_MN, acceptable models were obtained when a northern inland source was paired with either Bianbian or a source geographically further south (e.g., Fujian_EN or YR_MN). Notably, inclusion of either DHL/DCGL or Bianbian in the outgroup led to the rejection of most alternative models (data S8), supporting a proximal model for WLR_MN that includes both DHL/DCGL- and Bianbian-related sources. For Leigongshan and Zhengjiagou, inclusion of a DHL-related northern inland component in the outgroup led to the rejection of alternative models, supporting this component as the best-supported proximal source under the current modeling framework, although DHL could not be reliably distinguished from the other northern inland sources at the current resolution of qpAdm model fitting.

Furthermore, this DHL-related northern inland ancestry could also be incorporated as a more distal northern source for other later populations in qpAdm models, including ancient groups from the Yellow River Valley and the Tibetan Plateau, together with lineages such as Xingyi_EN or Fujian_EN (data S8 and fig. S9). However, these qpAdm results do not demonstrate that DHL represents the best proximal source for all these groups, nor do they resolve the exact demographic processes underlying these connections.

### Genetic portrait of early Neolithic populations related to millet cultivation

In addition, the high-coverage shotgun-sequencing dataset of DHL-M2 was used to track the emergence and dissemination pattern of adaptive sequence variants (data S9). In conjunction with previously reported data, we reconstructed the spatiotemporal distribution of an East Asian–specific variant [*EGLN1*-Asp4Glu (rs186996510 G > C)] that contributes to hypoxia adaptation (*35*). This mutation is located on the *EGLN1* gene, which encodes prolyl hydroxylase 2, a regulator of the hypoxia-inducible factor pathway sensitivity. It is associated with hemoglobin concentration in Tibetan populations and has been enriched through selection in high-altitude populations. Notably, among all ancient individuals with coverage at this site, only a few have the mutation. It was observed that all Late Pleistocene individuals (e.g., MZR, Tianyuan, AR-33K, AR-19K, and UKY) lack the derived allele. The first reported occurrence of this adaptive allele was in DHL-M2. Subsequently, the mutation is also present in the surrounding 7900–yr B.P. DCGL site and 7500–yr B.P. Siberian ancient individuals (Russia_Shamanka), as well as in one individual from Mongolian samples, indicating that it was present at low frequency in East central Asia during the Early Neolithic period. The mutation is also found in Bronze Age ancient individuals from the Tibetan Plateau (Fig. 4A). In modern populations, this mutation is primarily distributed among ethnic minority groups of the Tibeto-Burman language family, such as the Naxi and Yi, and exhibits a particularly high frequency in Tibetan populations, in contrast to a limited presence in the Northern Han Chinese population (Fig. 4B). Notably, an additional key mutation on *EGLN1* (rs12097901), although not observed in DHL-M2, was identified in both ST and DCGL (fig. S10). These genetic results suggest a possible early connection between Tibetan Plateau populations and inland nEA populations in the Neolithic period. However, another prominent hypoxia adaptation variant (*EPAS1*) exhibits a distinct pattern (*18*), as its adaptive alleles have not yet been detected in our DHL and ZJK individuals (data S9).

**Fig. 4.**
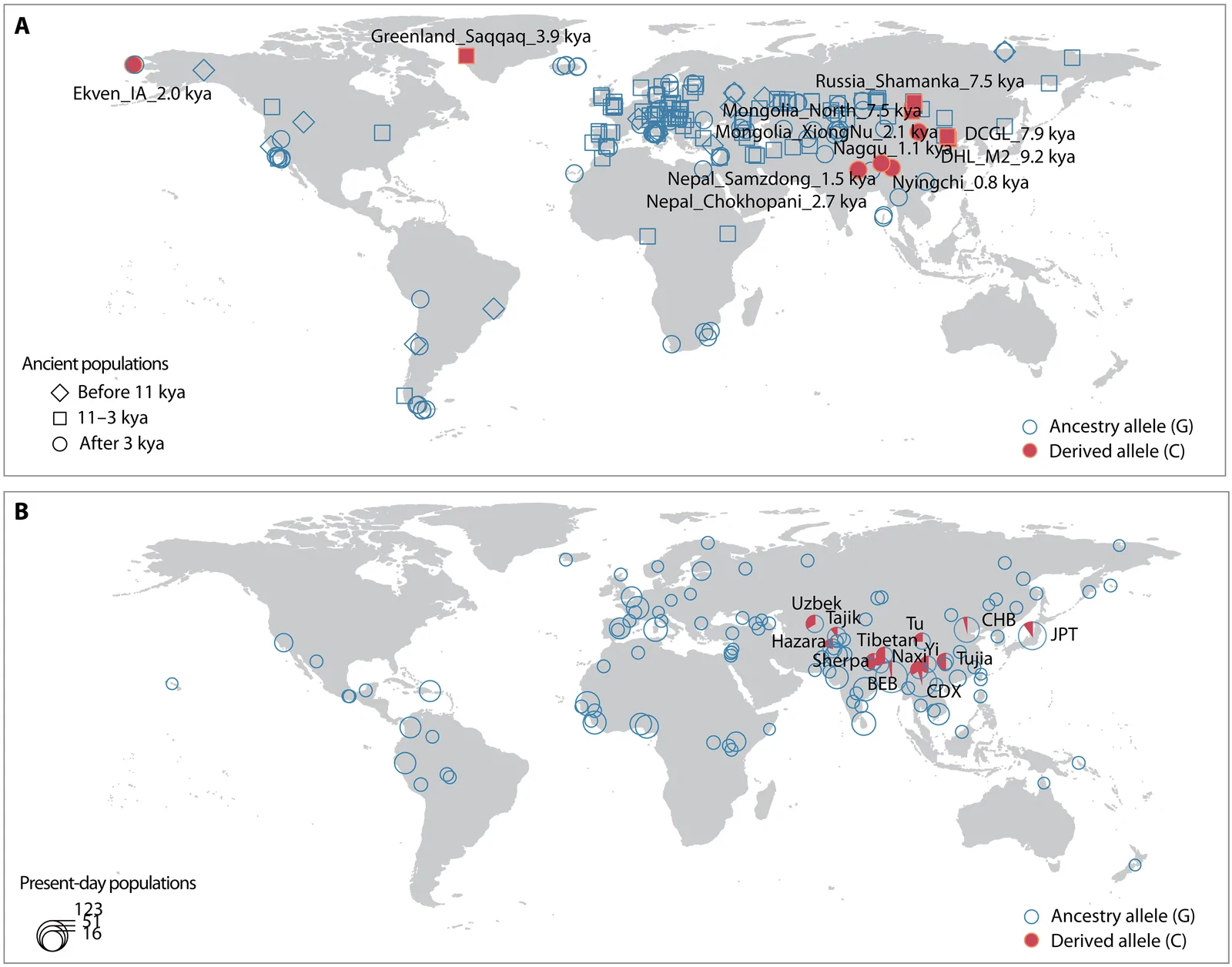
The spatiotemporal distribution of *EGLN1*-Asp4Glu. (**A**) The distribution of this variant in ancient worldwide individuals. Each point is a single individual. Individuals with this variant are marked by red with their name and age. DHL-M2 is the reported earliest ancient individual carrying this variant. (**B**) The distribution frequency of this variant in present-day worldwide populations shows that it is mainly distributed in Tibeto-Burman populations. The size of circle corresponds to the population size. Base map outlines were drawn using the world database in the R package maps through map (“world”).

We further reconstructed the appearance characteristics of DHL-M2, two DCGL, and two ST individuals. Analysis of pigmentation-associated traits with HIrisPlex-S indicated that all five individuals were most likely to have brown eyes and dark hair, with posterior probabilities exceeding 99.5 and 97%, respectively. For skin color, HIrisPlex-S assigned the highest posterior probabilities to the dark category for the DHL and ST individuals and to the dark-to-black category for the DCGL individuals (data S10). We also examined East Asian–associated pigmentation variants (*36*) as an additional qualitative assessment, applying the same scoring framework to present-day East Asian individuals from the 1000 Genomes Project (*37*) for comparison (data S10). The pigmentation polygenic scores of all five ancient individuals fell within or below the present-day East Asian distribution, with DHL-M2, DCGL-NL, and ST-F2-5L showing lower scores than the present-day mean, suggesting relatively darker genetically predicted pigmentation. Overall, these results provide preliminary insight into the appearance of individuals associated with early millet agriculture and should be considered as exploratory inferences rather than precise phenotypic assignments.

## DISCUSSION

Millet, as a key staple crop of dryland agriculture in nEA, profoundly affected prehistoric human subsistence and civilization across a large portion of East Asia and the wider Eurasian continent. In the northern China region, macrobotanical remains dating back 8800 to 7000 years have been unearthed at multiple archeological sites, spanning from southeastern Inner Mongolia to the northern inland region (*38*–*42*). Among them, the direct dating result of millet reported from the ST site is close to those of the Xinglonggou site in the West Liao River basin, which yields an age of approximately 7600 years (*43*), definitively demonstrating that the period around 8000 yr B.P. was critical for the expansion of millet agriculture. However, during the earlier period, evidence of macrobotanical millet with domesticated characteristics (14 carbonized foxtail and 1 carbonized broomcorn millet grain) has been recovered exclusively from the DHL site (*12*), and their age was inferred from radiocarbon dating of Phellodendron seeds from the same archeological unit (10,192 to 9766 cal yr B.P.). Stable carbon (δ^13^C) isotope results from DHL individuals also indicate the incorporation of millet food into subsistence strategies, following initial activities related to millet cultivation in the DHL area. Therefore, by comparing ancient genomes of DHL residents with those of later agricultural populations, the genetic origins of millet farmers in northern China can be revealed, whether they were immigrants or derived from local indigenous communities.

Our study contributes critical genome data from the DHL site and the nearby ZJK area (DCGL and ST sites), as well as isotopic and dating data. Genetic analysis shows no genetic differentiation among DHL individuals from different periods. We note that DHL-M1 and DHL-M2 in this study correspond to the same biological individuals as those recently reported for DHL_M1 and DHL_M2 (*44*). Although their study suggests that DHL_M1 represents a deeply diverged genetic lineage distinct from DHL_M2, our ancient DNA data more strongly support a close genetic continuity among all DHL individuals. This difference may reflect variations in data quantity and quality resulting from different DNA extraction, library construction, and sequencing processes, while the higher resolution of our ancient DNA data leads to more reliable conclusions (Supplementary Text, figs. S11 to S13, and table S1).

We further show that the genetic makeup of DHL individuals is most similar to DCGL and ST individuals, all belonging to the same genetic branch. This pattern suggests a notable degree of genetic continuity, indicating that the DHL and ZJK populations may represent some of the earliest groups inhabiting this northern inland region since the Early Neolithic period and subsequently initiated the millet agriculture. However, because some of the earliest DHL individuals already show evidence of C4 plant consumption and no local genomic data predating the period of millet use are currently available, whether the earliest millet consumers were genetically continuous with preceding local hunter-gatherers remains unresolved. Comparable genetic continuity has also been observed in the Fertile Crescent, another early center of agricultural origin (*45*, *46*). In contrast, evidence from Western Anatolia and Europe has shown that the spread and adoption of agriculture could involve different processes, including demic diffusion, cultural transmission, or a combination of both (*39*–*42*, *44*). These studies suggest that the relationship between the emergence and spread of agriculture and patterns of human mobility could vary considerably across regions. Notably, the situation in the southwestern area of North America also contrasts with that in Europe, where maize agriculture originated through intergroup transmission rather than from a long-distance migration of Mesoamerican farmers (*47*). Our findings are currently constrained by the available sample size, and future genomic investigations of relevant ancient populations will be necessary.

Tracking the spatiotemporal changes of mutations associated with human population phenotypes helps reconstruct prehistoric patterns of natural selection that shaped these adaptive events. Benefiting from the high-coverage genomic data obtained from DHL-M2 and four ZJK individuals in this study, we found that among all ancient individuals reported to date, a high-altitude hypoxia adaptation mutation (*EGLN1*-rs186996510 C > G) was first reported in DHL-M2 but at a lower frequency during the Early Neolithic. This mutation was also found in a few individuals in the surrounding regions of inland East Asia around 7500 years ago. More ancient DNA evidence is needed to determine when and through what route this mutation was subsequently transmitted to Tibeto-Burman groups and selected. However, this information also suggests about the potential link between the Early-to-Middle Neolithic nEAs and the ancestors of the Tibeto-Burman groups. In addition, we used multigene combinations to infer the physical appearance traits of DHL and ZJK individuals. Although the hair and eye color traits are broadly similar to those in present-day East Asian populations, skin-color inference should be interpreted more cautiously because current prediction models were not specifically optimized for East Asian populations. Our supplementary analysis nevertheless provides tentative support for relatively darker pigmentation in some of the ancient individuals compared with present-day East Asians. At the single-variant level, the East Asian–associated *OCA2*-615Arg variant (*48*), which is associated with lighter skin pigmentation, was not detected in the callable data from the individuals analyzed here. Previous hypotheses suggested that selection for lighter skin tone mutations occurred after the advent of agriculture, as the shift from a meat-based to a plant-based diet necessitated increased vitamin D synthesis (*49*). Our findings provide further evidence that the genetic mutations may not have arisen or become prevalent in the inland regions of northern China, at least by the early Middle Neolithic period.

In conclusion, our study reports high-quality genomic data from the population associated with the earliest millet cultivation in East Asia. Genetic analysis indicates that the DHL population represents a principal genetic component of the inland populations in nEA after the LGM and exhibits a close phylogenetic relationship with hunter-gatherer populations from the Amur River Basin. Furthermore, the DHL population is genetically highly similar to the 8000– to 7500–yr B.P. ZJK farmers (DCGL and ST). They all cluster within the same genetic branch, which strongly supports a local genetic origin model for millet agricultural populations in the region surrounding the DHL site. Further analysis also reveals that the genetic ancestry related to DHL had important impacts on the formation of later millet-farming populations. This study also identified the early appearance of a key high-altitude adaptation gene variant (*EGLN1* rs186996510) in the DHL and ZJK populations and reconstructed their physical phenotypic traits, providing insights into the process of human adaptation to agricultural life and environmental conditions.

## MATERIALS AND METHODS

### Archeological context

DHL is located on the third terrace of the Qingshui River in Beijing, whereas ST and DCGL are located in the ZJK region of Hebei Province, northern China. The skeletal remains analyzed in this study were recovered from archeological excavations or rescue-preservation work conducted at these sites by the relevant archeological institutions. The DHL samples were provided by Peking University and the Beijing Institute of Archaeology, the ST samples by the Hebei Provincial Institute of Cultural Relics and Archaeology and the Zhangjiakou Municipal Institute of Cultural Relics and Archaeology, and the DCGL samples by the Hebei Provincial Institute of Cultural Relics and Archaeology, Hebei Normal University, and the Zhangjiakou Municipal Institute of Cultural Relics and Archaeology. All sample identities and archeological contexts were verified by the excavating archeologists, and all necessary permissions for excavation, sampling, and analysis were obtained from the relevant local authorities and curating institutions.

### Radiocarbon dating

To prepare bone samples for analysis, surfaces were first cleaned using ultrasonic cleaning in distilled water, and then the bones were broken into smaller pieces. Acid-alkali-acid procedures (*50*) were followed to extract collagen from the bone. Specifically, 0.5 N HCl was used as the acid solution and 1 N NaOH as the alkali solution to treat the samples, followed by hydrolysis in a pH 2 to 3 HCl solution at 90°C for 24 hours. After centrifugation and lyophilization, gelatin was obtained from the samples.

The carbon and nitrogen content of the gelatin was analyzed using an Elemental Analyzer (Elementar, vario EL). All samples’ gelatin had a carbon-to-nitrogen ratio (C/N value) ranging from 2.9 to 3.6 (*51*). Samples were then weighed according to their carbon content and sealed in quartz tubes with copper oxide and silver under a vacuum system. The tubes were then combusted at 850°C to produce CO_2_, which was purified and transferred to a gas container. The CO_2_ was then reduced to graphite using a hydrogen-iron catalyst in our graphitization lines. The catalyst was first cleaned and activated at 450°C using oxygen and hydrogen separately. Graphite was formed at 540°C using magnesium perchlorate to trap water. Last, the graphite samples were sent to the AMS Center, School of Physics, Peking University (BA), for radiocarbon measurements. The accelerator mass spectrometry (AMS) system is based on a National Electrostatics Corp 1.5SDH-1 0.5-megavolt pelletron with a 40-sample MC-SNICS ion source. The accuracy of this system is better than 0.4%, and the machine background is lower than 0.03 pMC. The dates were calibrated using OxCal v.4.4.4 (*52*) with the IntCal20 atmospheric curve (*53*).

### Carbon and nitrogen stable isotope analysis

Carbon and nitrogen stable isotope measurements were performed using an Elemental Analyzer (vario PYRO cube) and Mass Spectrometer (IsoPrime100) at the Isotope Analysis Laboratory, School of Archaeology and Museology, Peking University. Carbon isotope results are expressed as δ^13^C, with Vienna Pee Dee belemnite as the standard reference material. Nitrogen isotope results are expressed as δ^15^N, with atmospheric nitrogen as the standard reference material. In this experiment, two international standard reference materials, IAEA-600 caffeine and USGS41 glutamic acid, were used for calibration via a two-point calibration method. The SD of the δ^13^C values obtained from laboratory internal replicate measurements was less than 0.10‰, and the SD of the δ*^15^*N values was less than 0.15‰. In addition, previously published comparative stable-isotope records from the Yumin, Xinglongwa, and Xinglonggou I datasets were compiled for comparative analyses and are summarized in data S4 (*23*, *54*–*56*).

### Ancient DNA processing

All human skeletal samples were obtained from the School of Archaeology and Museology at Peking University and processed in dedicated ancient DNA clean-room facilities at the Biomedical Pioneering Innovation Center, Peking University. Sample pretreatment, DNA extraction, and double-stranded library construction followed procedures previously described in Yang *et al.* (*21*) with modifications according to sample type.

Briefly, petrous bones were mechanically cleaned using disposable tools and sampled from the dense osseous region surrounding the cochlea. Auditory ossicles were processed without powdering and were decontaminated by sequential washing with 1% NaClO and 95% ethanol, followed by ultraviolet (UV) irradiation for 10 min on each side. For other skeletal elements, including femora and tibiae, adhering surface material was first mechanically removed. The samples were then sequentially washed with 4% NaClO, ultrapure water, and ethanol, followed by UV irradiation for 15 min before powder preparation. DNA was extracted from 50 to 100 mg of bone powder or from complete auditory ossicles using a silica-based protocol. A 30-min predigestion step was included to reduce surface-derived contamination, and at least one extraction blank was processed with each batch.

Double-stranded libraries were generated using a modified single-tube strategy based on the NEBNext Ultra II DNA Library Prep Kit for Illumina, following Yang *et al.* (*21*). The protocol incorporated enzymatic treatment to reduce cytosine-deamination–derived damage signals, followed by end preparation, Y-adapter ligation, first-round uracil-tolerant amplification using KAPA Uracil HotStart HiFi ReadyMix, and second-round indexed polymerase chain reaction amplification. Specifically, DNA extracts were treated with USER enzyme at 37°C for 50 min, followed by UGI treatment at 37°C for 30 min in end-repair buffer before end-prep enzyme was added to the same reaction. Final libraries were quantified using a Fragment Analyzer and sequenced on an Illumina NovaSeq platform with paired-end 2 × 150–base pair (bp) reads. Base calling and exact-index filtering were performed using CASAVA 1.8.2. Reads with an average Phred quality score or a Phred score across at least 50% of bases ≤ 20 were removed before downstream analyses.

### In-solution ancient DNA capture

RNA probes targeted to the whole-genomic “1240k” single-nucleotide polymorphism (SNP) panel and mtDNA were used for capture, performed at the Biomedical Pioneering Innovation Center at Peking University. The 1240k DNA probes, designed on the basis of prior publications, were synthesized and then reverse transcribed into RNA for use, while human mtDNA probes were purchased from iGeneTech (TargetSeq One Kit, lot TC1NTC2N). The mtDNA probes were diluted 100-fold and merged with the 1240k probes for simultaneous enrichment. Each enrichment used 500 ng of DNA library. The libraries were mixed with blocking oligos (Hyb Human Block and Universal Block from iGeneTech, compatible with Illumina TruSeq Libraries), and then biotinylated RNA probes (1240k and mtDNA), ribonuclease block, and hybridization buffer were added. The mixture was denatured at 85°C for 5 min and hybridized at 50°C for 24 hours. Following hybridization, streptavidin-capped beads captured the products for 30 min at room temperature. Unbound DNA was eliminated with Wash Buffer 1 at room temperature for 15 min, followed by three washes with TargetSeq One Wash buffer at 50°C for 10 min each. Subsequently, the beads were washed with ethanol and dried, and the captured libraries were amplified with P1-P2 primers for 12 cycles using Q5 High-Fidelity DNA Polymerase and purified by VAHTS DNA Clean beads. Last, libraries quantified by Fragment Analyzer were sequenced on an Illumina NextSeq 500 platform with paired-end 2 × 75 cycles (2 × 8 for the indices).

### Sequencing data analysis

First, adapter sequences were clipped, and paired-end sequencing reads were merged into a single sequence using AdapterRemoval. Merged reads longer than 30 bp were aligned to the human reference genome (GRCh37d5) using BWA-samse (v0.7.15-r1140) (*57*) with seed disabled parameters (-l 16500 -n 0.01 -o 2). Reads with a mapping quality below 30 were excluded using Samtools (v1.9) (*58*). Duplicate reads were subsequently removed using Picard markduplicates (v2.21.8) (http://broadinstitute.github.io/picard/). Then, uniquely mapped reads were selected for further analysis. For mtDNA, merged reads were aligned to the revised Cambridge Reference Sequence (rCRS) (*59*) using the BWA aln/samse algorithm. Duplicate reads were removed using DeDup 0.12.3 (https://github.com/apeltzer/DeDup), and consensus sequences were generated using Schmutzi (*60*). To prevent false SNP calls due to retained DNA damage, terminal damage patterns were calculated with mapDamage 2.0 (*61*). Then, the terminal 3 bp from both ends of reads were trimmed to remove the damaged signal based on the pattern. For each individual, pseudodiploid genotypes were generated using pileupCaller (from sequenceTools, v1.2.2), which selects a high-quality base (Phred-scaled base quality score ≥ 30) at random for each targeted SNP site (https://github.com/stschiff/sequenceTools). Following this, our genotypes were merged with those of previously published ancient individuals as listed in data S11 (*17*–*24*, *30*, *31*, *33*, *34*, *62*–*88*).

### Authenticity and contamination estimation of ancient DNA

To authenticate ancient DNA samples and estimate both authenticity and contamination, we used a multifaceted approach. Initially, we used mapDamage 2.0 to assess the terminal damage rate, with an expectation that a rate of ≥10% would indicate a potentially authentic ancient DNA signal (*89*). We then applied two criteria to estimate contamination rates. First, we used ContamMix (*90*) to compare mtDNA fragments of each individual with 311 mitogenome sequences from worldwide populations (using 3-bp terminal trimmed BAM files), treating libraries as contaminated if more than 5% of the fragments matched one of the worldwide sequences better than the consensus. Second, we evaluated the nuclear genome contamination rate for males based on X-chromosome data using ANGSD v0.920 (*91*), which accounts for sequencing errors and mismatches on polymorphic positions, considering libraries as contaminated if the estimated contamination rate exceeded 5%.

### Sex determination and uniparental haplogroup assignment

For sex determination and further analysis of uniparental genetic markers, we assessed the genetic sex of our ancient samples by analyzing the ratio of X- and Y-chromosome coverages relative to autosomes. Consensus mtDNA sequences were generated using Schmutzi, and the resulting FASTA files were used to determine mtDNA haplogroups with PhyloTree Build 17 (*92*) against the rCRS. To infer Y-chromosome haplogroups, we first extracted uniquely mapped reads aligned to the hg38 reference genome using BWA-MEM. We then compiled a reference panel of 32,656 Y-chromosome SNPs from an up-to-date Y-chromosome phylogenetic tree (http://yoogene.com/sourse/) and genotyped each individual at these loci. Expected allele profiles for all haplogroups in the tree were reconstructed from the ancestral and derived states of their defining SNPs. For each sample, genotype calls supported by at least one read and without heterozygous signals were retained and compared with the expected profiles across haplogroups. The final haplogroup assignment was based on the highest similarity score between the observed and expected SNP profiles.

### Principal components analysis

PCA was conducted using the smartpca program (version 13050) from the EIGENSOFT package (version 7.2.1) (*93*). Eigenvectors were constructed using the present-day populations listed in fig. S3A. The ancient individuals’ positions were determined through projection using the lsqproject: YES option. Other options were set to default except for numoutlieriter: 0 and autoshrink: YES.

### *F* statistics

We applied outgroup-*f*_3_ statistics [*f*_3_(Test, DHL; Mbuti)] to assess the shared genetic drift of DHL with other Eurasian populations, using Mbuti as the outgroup. Furthermore, to determine whether a test population is symmetrically related to populations *X* and *Y*, *f*_4_ statistics [*f*_4_(Mbuti, Test; *X*, *Y*)] were computed using the qpDstat program from ADMIXTOOLS 4.1 (*94*) with the parameter *f*_4_ mode: YES. The Mbuti population was designated as an outgroup for all *f*-statistics analyses. The aforementioned statistics are reported when more than 30,000 SNP positions overlapped between the four analyzed populations. SEs in *f*_4_ statistics were calculated from the default block jackknife approach. *F*_3_ and *F*_4_ statistics were computed using qp3Pop v435 and qpDstat v755 in the ADMIXTOOLS 4.1 package.

### Admixture modeling with qpAdm and qpGraph

We used qpAdm (v650) from ADMIXTOOLS 4.1 with the parameter “allsnps: No” to identify the most probable ancestral sources and model the ancestries of admixed populations. The analysis incorporated autosomal SNPs from the 1240k dataset. We established a base set of outgroups to model the admixture history of inland nEA populations and their relationship with DHL. This panel consisted of representative populations from major global lineages: Mbuti.DG, Onge.DG, Anatolia_Neolithic, Tarim_EMBA1, AR33K, Kolyma_M, Fujian_LN, Villabruna, AR19K, and Japan_Jomon (data S6). All reference combinations were tested by qpWave (v400) to ensure that they represented distinct sources under these outgroups.

In addition, we proceeded to assess mixture models for younger agriculture-related groups. A proximal model combined three main possible sources: Yunnan Basal Asian (Xingyi_EN) and ancient southern East Asian (Fujian_EN). This model was applied to target ancient Tibetan populations, ancient inhabitants of the Yellow River Valley, and Central Plains of China. We progressively tested one-, two-, and three-way mixture models until viable models (*P* > 0.05) were identified. Different outgroup population sets were used for various regions, with detailed models available in data S8.

We constructed a phylogenetic tree model to satisfy conditions that aligned with the *f*_2_, *f*_3_, and *f*_4_ values between populations and individuals using qpGraph (*95*), with the allsnps: yes parameter. We sequentially incorporated each population into the model. For each target population, we considered its origin from either a single ancestral line or a mixture of two distinct tree branches.

### Phenotypic traits analyses

We examined the presence of alleles related to biological traits that could be of interest in the geographical and temporal context of the reported ancient populations, including lactase persistence (*96*, *97*), hypoxia resistance (*35*), alcohol metabolism (*98*), and skin pigmentation (*99*–*101*). The allele distribution for the SNP positions listed in data S9 was tabulated for each individual using samtools mpileup (v1.9).

Pigmentation phenotypes of hair, skin, and eyes were predicted with the HIrisPlex-S web tool (*102*, *103*) for each of the sequenced samples. In case of missing genotypes for any of the 41 SNPs used by HIrisPlex-S, sites with low or no depth in the ancient dataset were examined directly in the BAM files, and the most abundant allele was chosen (data S10). In BAMs, we only considered bases at least three bases away from either end of the read, with a base quality ≥ 25, and no C-to-T and G-to-A SNPs to avoid any effect of postmortem damage on the prediction.

In addition, we performed a supplementary qualitative check based on 26 previously reported lead variants associated with skin color variation (*36*). For each sample, callable SNPs among these 26 sites were used to calculate a simplified L*-weighted score (pigmentation polygenic scores) based on the reported effect sizes. The same scoring framework was applied to present-day East Asian individuals from the 1000 Genomes Project (*37*) for comparison. Individual-level scores and population summary statistics are provided in data S10.

### Spatial-temporal tracing of hypoxia adaptation–related variants

We examined the spatial-temporal distribution of hypoxia adaptation–related variants, specifically *EGLN1*-Asp4Glu(rs186996510 (G > C), using a worldwide ancient DNA database (e.g., the Allen Ancient DNA Resource: https://dataverse.harvard.edu/dataset.xhtml?persistentId=doi:10.7910/DVN/FFIDCW) and collected modern human population data (data S10; excluding samples with missing genotypes) to construct the spatial-temporal distribution map of the variants. Our analysis revealed that DHL-M2 is the earliest known individual carrying this mutation.

## Supplementary Materials

### The PDF file includes:

Supplementary Text Figs. S1 to S13 Table S1 Legends for data S1 to S11 References
