# Supplementary material for: Early Neolithic genomes suggest a local origin for millet farmers in East Asia
Source: Sci Adv. 2026 Oct 16;12(42):eaef9182. doi: 10.1126/sciadv.aef9182 (PMC13674475; doi:10.1126/sciadv.aef9182)
Supplement: Supplementary file 1 [file sciadv.aef9182_sm.pdf]

Science Advances
AAAS

Supplementary Materials for

Early Neolithic genomes suggest a local origin for millet farmers in East Asia

Tingyu Yang *et al.*

Corresponding author: Yanyi Huang, yanyi@pku.edu.cn; Yuhong Pang, pangyuhong@pku.edu.cn; Chaohong Zhao, zhaoch@pku.edu.cn; Xiaohong Wu, wuxh@pku.edu.cn

Sci. Adv. **12**, eaef9182 (2026)
DOI: 10.1126/sciadv.aef9182

The PDF file includes:

Supplementary Text
Figs. S1 to S13
Table S1
Legends for data S1 to S11
References

Other Supplementary Material for this manuscript includes the following:

Data S1 to S11

Supplementary Text

Provenance statement

All skeletal remains examined in this study were recovered from archaeological excavations. The archaeological context of the skeletal remains is summarised below and described in detail in "Archaeological sites background".

Two Donghulin samples, DHL-M1 and DHL-M2, derive from well-documented archaeological excavations conducted at the Donghulin site by Peking University and the Beijing Institute of Archaeology in 2003 and 2005, respectively. DHL-M3 was collected by the Beijing Institute of Archaeology in 2016 during a rescue-preservation operation at the Donghulin site after rainwater erosion exposed the skeletons. Individual DHL-M01 was discovered and collected at the Donghulin site during a field survey conducted by Peking University in 1995, while DHL-M02, which may derive from the same individual as DHL-M01, was discovered and collected during archaeological excavations conducted at the Donghulin site by Peking University and the Beijing Institute of Archaeology in 2001.

The Sitai samples derive from well-documented archaeological excavations conducted at the Sitai site by the Hebei Provincial Institute of Cultural Relics and Archaeology and the Zhangjiakou Municipal Institute of Cultural Relics and Archaeology between 2020 and 2022.

The Dengcaogouliang samples derive from well-documented archaeological excavations conducted at the Dengcaogouliang site by the Hebei Provincial Institute of Cultural Relics and Archaeology, Hebei Normal University, and the Zhangjiakou Municipal Institute of Cultural Relics and Archaeology between 2015 and 2021.

The authenticity of the samples was confirmed by the archaeologists who excavated the sites. The archaeological dates of the burials were also established by the excavating archaeologists and are consistent with the radiocarbon dates, as detailed in Supplemental Data S1.

All skeletal remains from the Donghulin site are currently housed at the Tongzhou Base of the Beijing Institute of Archaeology. They can be accessed using the sample ID numbers provided in this study, subject to permission from Professor Chaohong Zhao, the archaeological field director, and approval from the relevant authorities of the two institutions involved in the excavation, namely Peking University and the Beijing Institute of Archaeology, in accordance with the appropriate institutional procedures.

All skeletal remains from the Sitai site are currently housed at Liaoning University and will be returned to the Hebei Provincial Institute of Cultural Relics and Archaeology. They can be accessed using the sample ID numbers provided in this study upon written

application to the Hebei Provincial Institute of Cultural Relics and Archaeology, with Tao Tong serving as the contact person.

All skeletal remains from the Dengcaogouliang site are currently housed at Liaoning University. They can be accessed using the sample ID numbers provided in this study upon application to the Hebei Provincial Institute of Cultural Relics and Archaeology and with permission from Yingjie Cui.

The open-access publication of the results of this study ensures unrestricted access to the results by specialists as well as the general public.

All materials analysed in the present study were provided by archaeologists who are also authors of this study. All necessary permits for the excavation and analysis of the ancient human remains were obtained from the relevant local authorities. We ensured that all samples were ethically sourced, and that the excavation, handling, and analysis of the human remains followed appropriate archaeological and bioethical guidelines, including procedures intended to minimise contamination and ensure respectful treatment of the remains. No export permission was required, as all genetic analyses and sample transfers were conducted within a single country.

Archaeological sites background

1.1 Donghulin (东胡林)

1.1.1 Site description

The Donghulin Site (*29*) (N 39.97, E115.72) is located on the third terrace of the north bank of the Qingshui River, west of Donghulin Village, about 78 kilometers away from the urban area of Beijing. The site is at an altitude of 390-400 meters, 25 meters above the current riverbed, and covers an area of about 3,000 square meters. From 2001 to 2006, the Donghulin archaeological team conducted four formal excavations on the site, and another rescue excavation was carried out in 2017. The excavation area is concentrated in the southwest part of the site, with an exposed area of over 200 square meters. Carbon dating was performed on various specimens collected from the Donghulin site, incluing wood charcoal, human and animal bones, pottery shards. The results show that they were dated to about 11,000 to 9,000 years ago. In addition to the rich cultural relics unearthed, tombs, ash pits, and fire pits were also discovered. More than ten fire pits were found, most of which were irregular circular in shape, generally with a diameter of 0.5–1 meter and a depth of 0.2–0.3 meters. The fire pits were usually filled with piles of stones in varying sizes, animal bones, and ashes.

The unearthed relics include stone tools, pottery, bone tools, shell tools, as well as a large number of stones and debris, and animal and plant remain. In addition, two

complete human bones were also excavated. Among the stone tools, the majority are chipped stone tools, followed by microliths, and the number of ground stone tools is relatively small, mainly small axes, adzes, and several grain processing tools such as stone grinding plates and rods. The pottery was all broken into pieces, with more than sixty in total. The surface of the pottery shards is generally reddish brown or grayish brown, and most of them are soft in texture due to the low firing temperature. The pottery shards can be restored to a flat-bottomed, straight-bellied pot with a thickened rim and stamped decoration. There are many types and large amount of bone tools, all made of animal limb bones, finely processed and polished smoothly. Among them, a bone handle knife embedded with a stone blade was found. The shell tools discovered are mainly decorative items made from clam or snail shells, possibly used as necklaces or pendants.

A relatively rich amount of plant remains were unearthed at the site (*12*), including four types of specimens collected by flotation: carbonized wood chips, seeds, fruit pits, and fruits. Among them, the most important are the seeds of the Poaceae family, from which two cultivated grains, foxtail millet and broomcorn millet, as well as two types of weeds, Poa sphondylodes and Setaria viridis, were identified.

1.1.2 Burial description

At least seven human remains have been recovered at the Donghulin site, three of which were excavated in the 1960s, but due to the loss of information about their storage location, no bone samples were obtained (*107*). The remaining individuals have been unearthed successively since 1995. Among them, DHL-M1 and DHL-M2 were excavated in 2003 and 2005, respectively, from two vertical pit tombs with clearly defined stratigraphic location. The human skeletons inside the toms are relatively complete and well preserved (*108*). The burial style of DHL-M1 features a single person lying supine with extended limbs, while DHL-M2 features a single person lying supine with flexed limbs. Individual DHL-M3 was discovered in 2006 in a section with the similar stratum to DHL-M1, but was not excavated due to limitations at the time. In 2016, rainwater erosion exposed some skeletal remains of DHL-M3, then Beijing Institute of Archaeology carried out rescue preservation and collected bone samples, including ribs, vertebrae, and clavicles from DHL-M3. Individual DHL-M01 and DHL-M02, each consisting of half a human skeleton, were discovered and collected in 1995 and 2001, respectively. Their stratigraphic contexts and burial practices are unclear, and morphological assessment does not allow confirmation of whether they derive from the same individual.

In this study, we screened skeletons of all the remaining individuals. DHL-M01 did not

pass the overall aDNA quality control, and DHL-M02 did not pass the nuclear aDNA quality control. We therefore included three individuals, DHL-M1, DHL-M2 and DHL-M3, to retrieve genome-wide aDNA data.

- DHL-M1: 11,174–10,696 calBP
- DHL-M2: 9,275–9,020 calBP
- DHL-M3: 10,805–10,504 calBP
- DHL-M02: 9,968–9,400 calBP

1.2 Sitai （四台）

1.2.1 Site description

The Sitai site (N 41.29, E 114.23) is situated on the terrace of a small lake, approximately 35 km northeast of Shangyi County, Zhangjiakou City, Hebei Province. It lies on the southeastern Inner Mongolian Plateau in northern China, with an altitude of around 1,450 meters above sea level (ASL). The surrounding vegetation is primarily typical steppe, featuring Stipa and Leymus chinensis steppes (*39*). The regional climate is characterized as cold and dry, with an annual mean temperature of 2.8°C and an annual mean precipitation of 397 mm (*109*).

The site covers an area of about 150,000 square meters. Recognized in 2004, excavations were conducted from 2015 to 2018 and again from 2020 to 2022. Two comprehensive excavation reports have been published for these periods(*39, 110*). Before 2020, four Neolithic semi-subterranean houses were discovered. By 2022, more than 40 dwellings had been identified, along with numerous artifacts including pottery, stone tools, bone fragments, and shells.

The archaeological features are classified into five groups. The first group (dating to 10,400–10,000 BP), second group (9,400–9,000 BP), and third group (7,600–7,400 BP) represent the Sitai culture, recognized as the earliest Neolithic culture in the region. The third group provides early evidence of dryland agriculture, featuring numerous stone grinding plates, grinding sticks, and other processing tools, as well as stone shovels and digging implements. Attributes from the fourth group (7,200–7,000 BP) belong to the late Yumin culture Phase II (Xinglong culture Phase II), marking the southern boundary of the Yumin culture. The fifth group (6,800–6,400 BP) reflects exchanges with northern cultures, offering crucial insights into the formation of the early steppe Silk Road.

The pollen record from the Sitai profile indicates a significant increase in trees during 8,000–7,000 cal BP, signifying the development of a deciduous broadleaved forest-

steppe vegetation landscape around the site and a more humid climate. The $\delta^{13}C$ values of human bones from the Sitai site, dated to approximately 7,700–7,600 cal BP, range from −12.3‰ to −13.6‰, suggesting a substantial proportion of C4 plants in the human diet (*39*).

1.2.2 Burial description

A total of 20 individuals have been identified from three rectangular dwellings at the Sitai site, all of which date to the third group (7,600–7,400 BP). Multiple burial positions and practices have been observed, including supine burial with extended limbs, flexed burials in a lateral position, and secondary burials. Two individuals were found in dwelling 2015F1, one buried supine with extended limbs and the other in a flexed lateral position. Five individuals were found in dwelling 2015F3, including two buried supine with extended limbs, two in a flexed lateral position, and one too fragemented for burial position to be determined. The dwelling 2020F2 contained 13 human skeletons. The skeletons were buried in disarray, with some deposited atop others. Evidence suggests that some individuals were buried with flexed limbs, and secondary burial may also be represented, as some individuals were preserved only as skulls.

In this study, we included two individuals from dwelling 2020F2 with sufficiently well-preserved skeletal remains, ST-F2-1 and ST-F2-5, to retrieve genome-wide aDNA data. The burial positions of these two individuals remain unclear. Radiocarbon dating indicates that the dwelling was occupied around 7,572 to 7,165 years ago, with a 95.4% confidence interval. The individuals who died earlier were primarily buried in the southeastern part of the house, while those who died later were interred in the western part of the house, near the center. ST-F2-5 belongs to the early group, whereas ST-F2-1 belongs to the later group.

- ST-F2-1: 7434–7335 calBP
- ST-F2-5: 7502–7425 calBP

1.3 Dengcaogouliang （邓槽沟梁）

1.3.1 Site description

The Dengcaogouliang site (N 40.87, E 114.93) is situated on a secondary platform on the east side of the Qingshui River valley, within the village of Dashuiggou in Chongli County, Zhangjiakou City, Hebei Province. It is located about 2 kilometers west of the city center of Zhangjiakou. Excavations of the site began in 2015 and cover an area of approximately 400,000 square meters. The site dates back between 8,000 and 4,000 years and has undergone four stages of cultural development.

In the first stage, about 8,000 years ago, one single house F3 was discovered that featured a square stone hearth at its center and indicated human burials on the floor. Artifacts unearthed from this stage include undecorated cylindrical jars, stone hoes with shoulders, round grinding stones, and stone balls, reminiscent of the Sitai culture. In the second stage, approximately 6,500 years ago, five houses were revealed. Artifacts from this period include red-painted pots, undecorated jars, and various other relics, aligning with the Hougang I period culture. In the third stage, around 5,100 years ago, another house was uncovered along with artifacts such as pottery bowls, painted pottery, and pottery with engraved patterns. This stage is similar to the Haishengbulang culture found in the western part of the great bend of the Yellow River. In the fourth stage, roughly 4,000 years ago, a stone-walled city was found featured with the Longshan Culture period.

1.3.2 Burial description

Two individuals, DCGL-NL and DCGL-SR, were recovered from House F3, dated to the first stage, and both were buried on the floor of the dwelling. DCGL-NL was buried in a lateral position with extended limbs, whereas DCGL-SR was buried in a lateral position with flexed limbs.

In this study, we retrieve genome-wide aDNA data from these two individuals.

- DCGL-NL: 7864–7688 calBP
- DCGL-SR: 7972–7752 calBP

<u>Comparative analysis of the Donghulin genomic datasets</u>

To investigate the source of the discrepancy between Zhang's claim (*44*) and our results in DHL-M1 and DHL-M2, we compared the datasets from both studies using multiple approaches, including pairwise mismatch rate, deaminated-read-based analyses, contamination assessment, coverage-matched downsampling, PCA, outgroup-f3 statistics and f4-statistics. To make the comparison clearer, we denote the data from Zhang et al (*44*) as Zhang_DHLM1 and Zhang_DHLM2, while retaining DHL-M1, DHL-M2 and DHL-M3 for our own dataset. To strictly eliminate the influence of modern contamination, we extracted deaminated reads (retaining only sequences with characteristic aDNA damage patterns) for all samples, labeling them with a "_d" suffix (i.e., Zhang_DHLM1_d, Zhang_DHLM2_d, DHL-M1_d, and DHL-M2_d).

First, we calculated the pairwise mismatch rate (PMR) to directly test whether the M1 and M2 reported in both studies are respectively the exact same individuals. Generally, identical individuals exhibit a PMR of 0.10–0.12, whereas first-degree relatives show a PMR of approximately 0.17. For M2, all combinations of our data with those reported

by Zhang et al (*44*) yielded PMR values of ~0.11. This proves that they are the exact same individual and indicates no severe contamination in either M2 dataset (Table S1).

However, a differnet pattern emerged for M1. When comparing DHL-M1 with Zhang_DHLM1, the PMR value (0.14056) falls between the expected range for the identical individual (0.10–0.12) and first-degree relatives (~0.17). Crucially, when we specifically utilized their deaminated data (Zhang_DHLM1_d), the PMR values of ~0.11 pointed to the identical individual, regardless of whether our own data was restricted to deaminated reads or not (Table S1). This indicates that Zhang_DHLM1 may suffer from a certain extent of modern DNA contamination. We therefore estimated contamination in the datasets of Zhang et al (*44*) using AuthentiCT (*111*), a statistical method designed for non-UDG-treated single-stranded ancient DNA libraries. The estimated contamination exceeded 10% in Zhang_DHLM1, whereas no comparable contamination signal was detected in Zhang_DHLM2.

Second, our DHL-M1 dataset covered approximately 2.5-fold more 1240K SNPs than Zhang_DHLM1 (426,222 versus 169,993 SNPs). To test whether this disparity in data coverage caused the divergent genetic results, we randomly downsampled our data to create a coverage-matched dataset, labelled as DHL-M1_downsample (174,385 SNPs), for subsequent comparative analyses.

In PCA, all DHL individuals from both studies cluster together, supporting their overall genetic similarity. Although our higher-coverage DHL-M1 and Zhang_DHLM1 show a slight positional difference, DHL-M1_downsample projects close to Zhang_DHLM1 (fig. S11). This spatial proximity confirms that the minor disparity in the PCA is likely a projection artifact driven by the difference in data coverage, rather than a true divergence. Outgroup-f3 statistics provided consistent results. Across a broad panel of test populations, both Zhang_DHLM1 and Zhang_DHLM1_d showed their highest shared genetic drift with DHL-M2, DHL-M3, and Zhang_DHLM2 (fig. S12), further supporting genetic affinity among the DHL individuals.

We next performed f4-statistics to formally test whether M1 and M2 form a genetic clade. We evaluated four M1–M2 pairings involving DHL-M1, DHL-M1_downsample, DHL-M2, Zhang_DHLM1, and Zhang_DHLM2. In tests of the form f4(Mbuti, Test; M1, M2), nearly all statistics were consistent with zero (fig. S13A–D). All comparisons involving our DHL-M1 or DHL-M1_downsample and DHL-M2 were consistent with symmetry across the full set of test populations (fig. S13A, B). The comparison between Zhang_DHLM1 and Zhang_DHLM2 was also predominantly consistent with symmetry, with only three test populations producing significantly non-zero values (fig. S13C).

We further evaluated their relative affinity using f4(Mbuti, M1; Test, M2) (fig. S13E–H). The higher-coverage DHL-M1 dataset showed strong genetic affinity with DHL-M2 (fig. S13E), whereas the statistical signal was reduced after DHL-M1 was downsampled (fig. S13F), demonstrating that lower SNP coverage decreases the power

of these tests. The results obtained with DHL-M1_downsample closely resembled those involving Zhang_DHLM1 and either Zhang_DHLM2 or DHL-M2 (fig. S13G, H). Even with this reduced statistical power, Zhang_DHLM1 showed greater affinity to Zhang_DHLM2 than to more than two-thirds of the test populations, which is inconsistent with its placement as a deeply divergent lineage (fig. S13G). Furthermore, we examined f4(Mbuti, M2; Test, M1), the configuration used by Zhang et al (*44*) to support divergence between M1 and M2 (fig. S13I–L). Our DHL-M2 and DHL-M1 datasets showed a strong relative affinity (fig. S13I). This signal again became weaker when DHL-M1 was replaced by the coverage-matched DHL-M1_downsample dataset (fig. S13J), confirming the effect of reduced SNP coverage on statistical power. The weaker signals obtained from Zhang_DHLM1 and Zhang_DHLM2 are therefore consistent with the lower coverage and elevated contamination of Zhang_DHLM1 rather than requiring deep genetic divergence between the two individuals (fig. S13K).

Taken together, these results do not support the interpretation of Zhang_DHLM1 as a deeply divergent lineage. Instead, the discrepancy between the two studies is more parsimoniously explained by modern DNA contamination and lower SNP coverage in Zhang_DHLM1, both of which reduce the reliability and statistical power of population-genetic analyses.

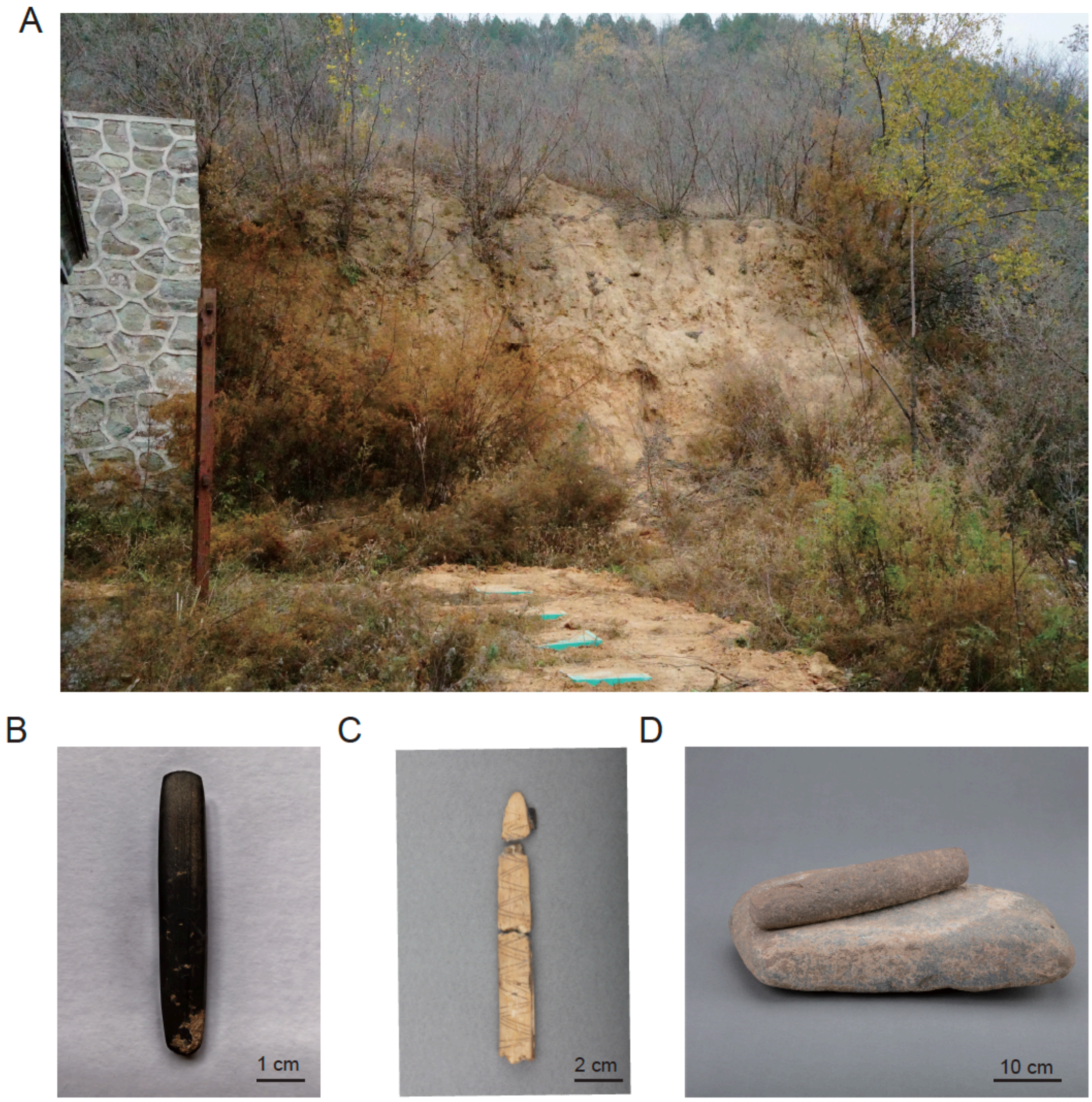

fig. S1. Archaeological background of the DHL Site. (**A**) Photograph of loess profile. (**B, C, D**) Artifact assemblages from the DHL site. (**B**) Ground stone adze (石锛); (**C**) stone blade with boned-handle (骨柄石刃刀); (**D**) stone milling slab and rolling pestle (石磨盘和石磨棒). (Photo Credit: C.Z., Peking University).

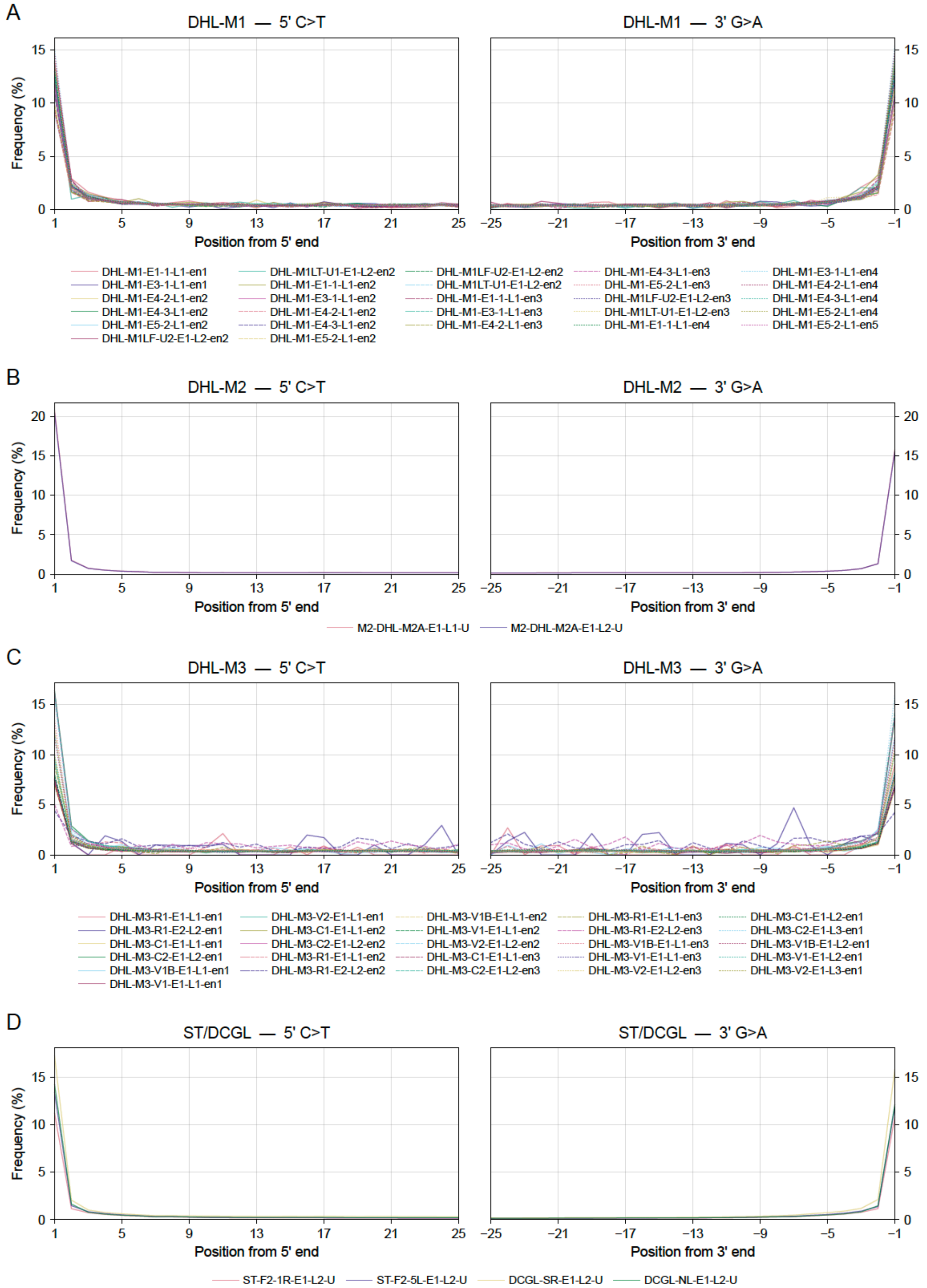

fig. S2. The damage patterns (C>T and G>A misincorporation levels) of all libraries. (**A**) All libraries for DHL-M1. (**B**) All libraries for DHL-M2. (**C**) All libraries for DHL-M3. (**D**) All libraries for ST and DCGL.

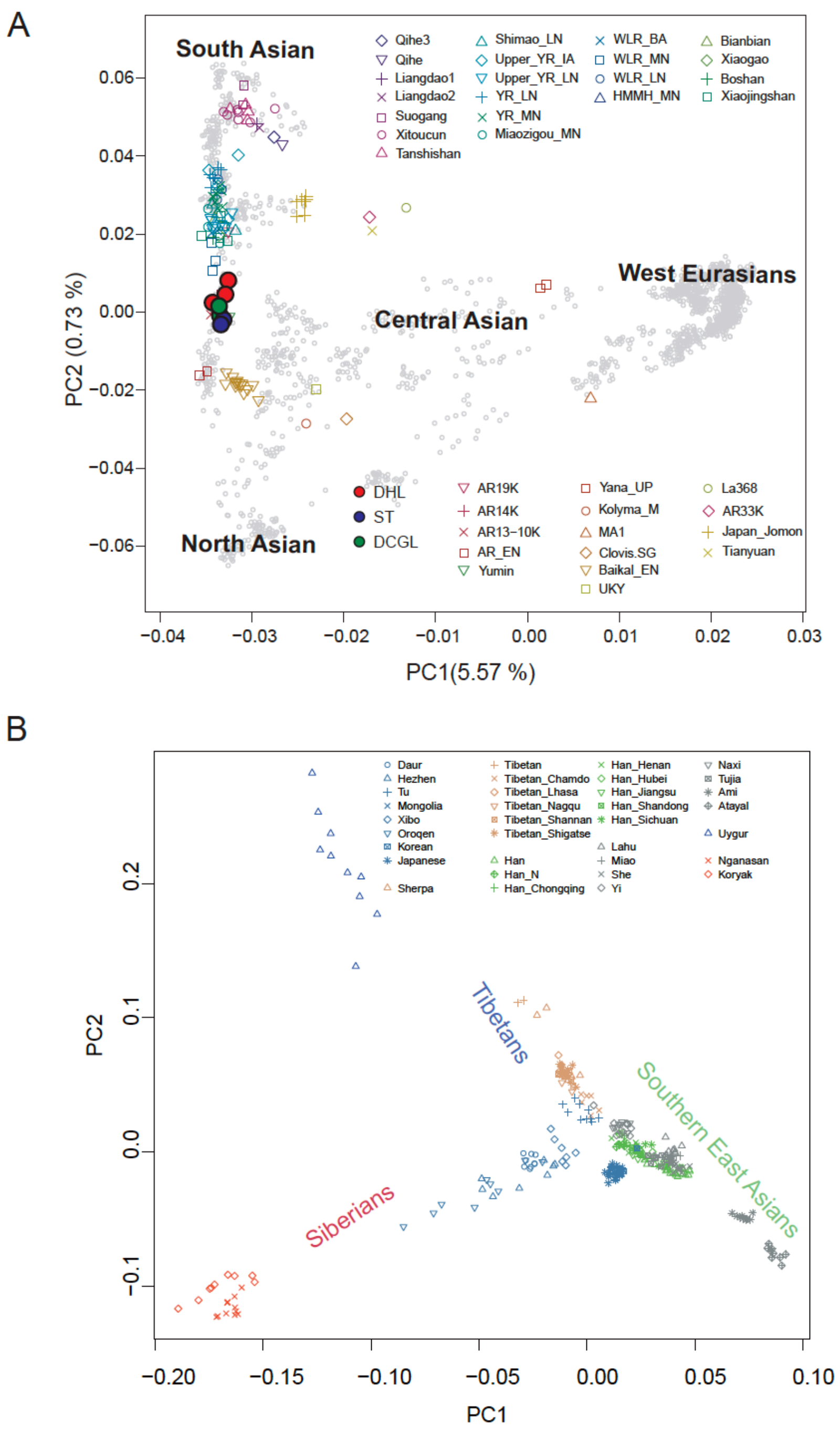

fig. S3. Genetic structure of analyzed populations. (**A**) Ancient populations are projected onto the principal components constructed from present-day Eurasians. Newly analyzed samples are highlighted by solid and larger symbols. (**B**) PCA for present-day East Eurasian populations corresponding to the background plot in Figure 2A. The analysis reveals a geographically correlated structure, characterized by three distinct trajectories, which correspond to Southern East Asia, Siberia and Tibet Plateau.

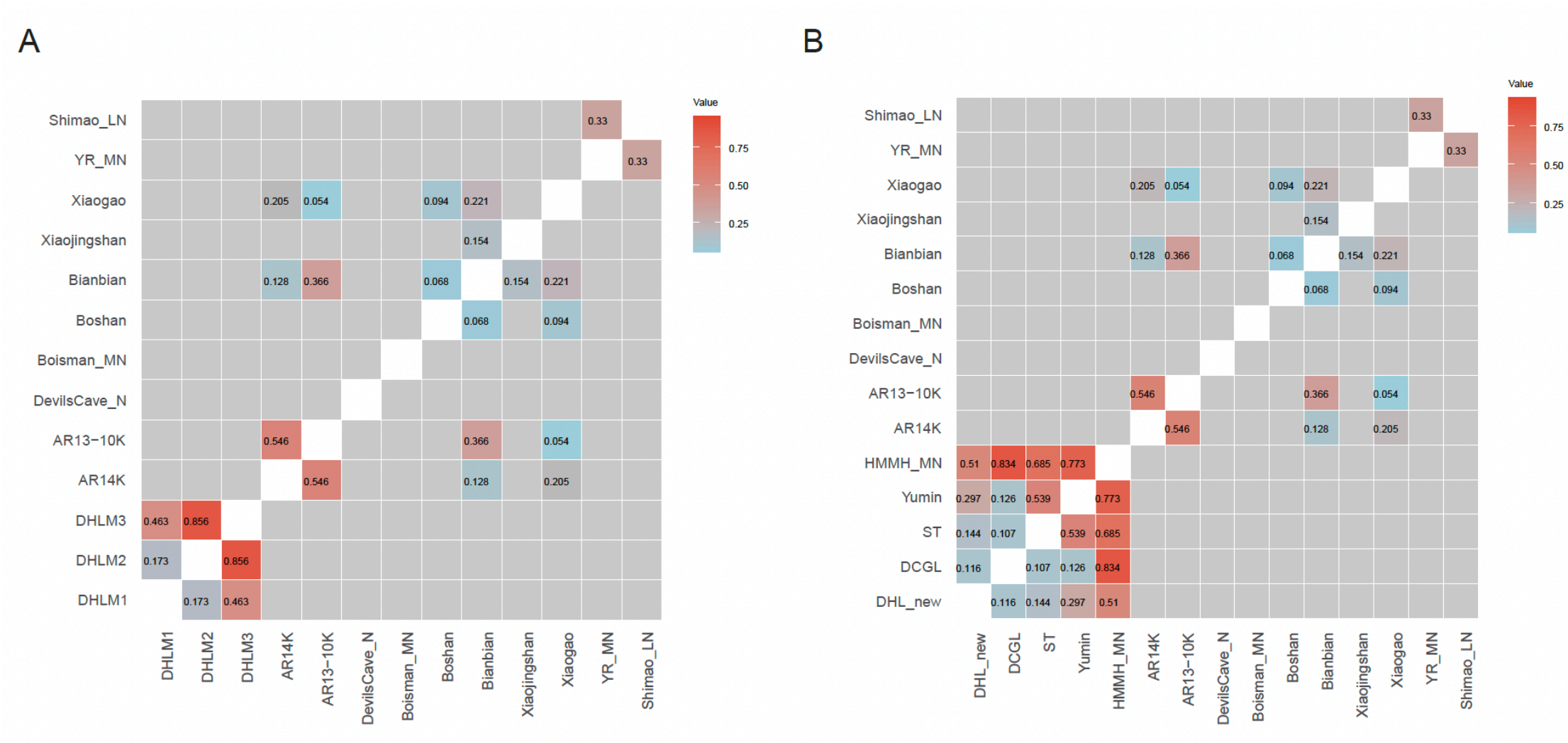

fig. S4. qpWave heatmap between each pair of northern East Asian populations. (**A**) Our newly sampled individuals from the DHL site are cladal with each other. (**B**) These inland northern East Asian groups (DHL, DCGL, ST, Yumin and HMMH_MN) are also cladal with each other.

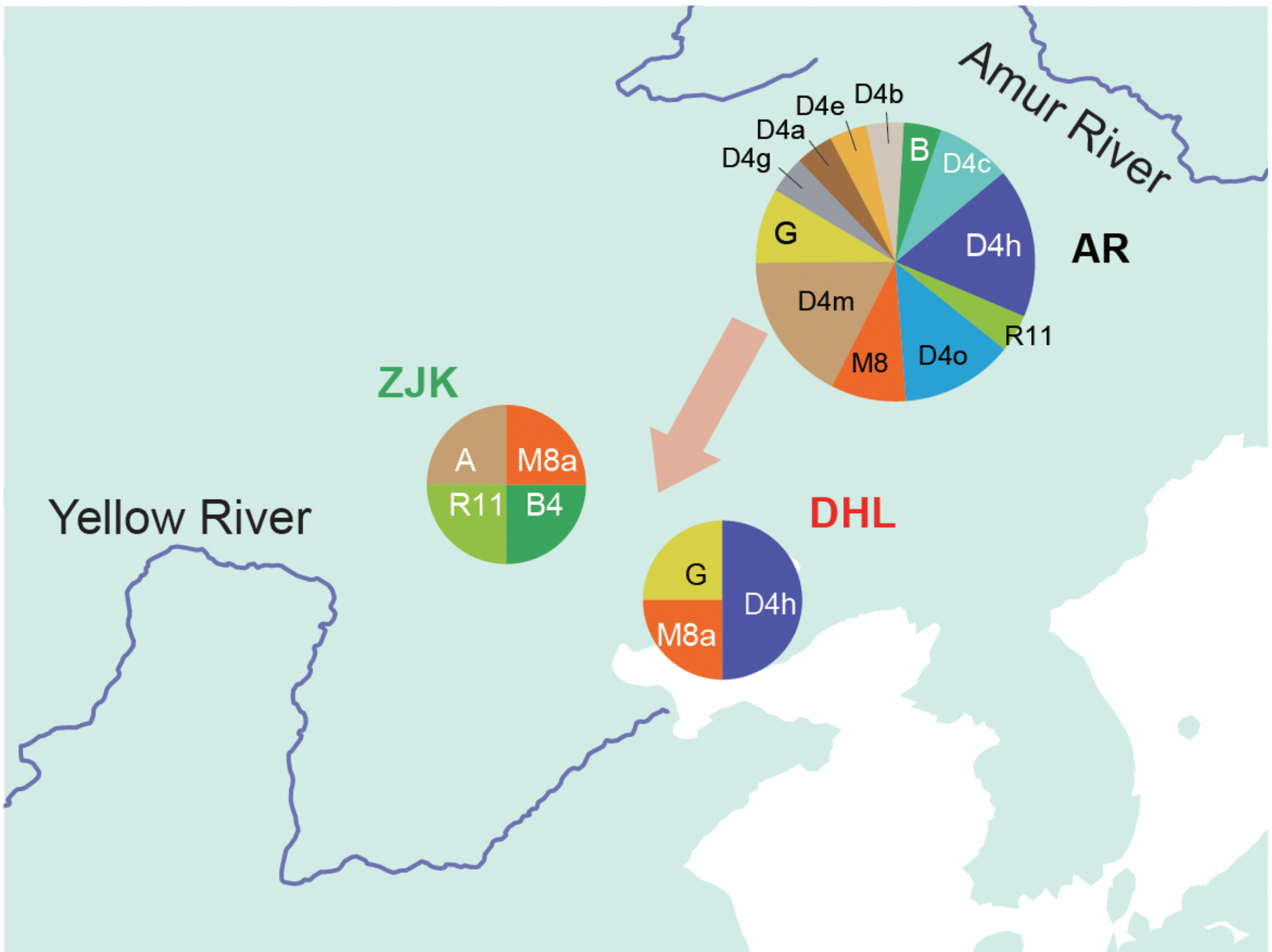

fig. S5. Distribution of mitochondrial haplotypes in DHL, ZKJ and Amur River basin. The mitochondrial haplotypes of DHL and ZJK (DCGL and ST) samples fall within the diversity of ancient populations in Amur River basin. Base map outlines were drawn using the world database in the R package maps through map("world").

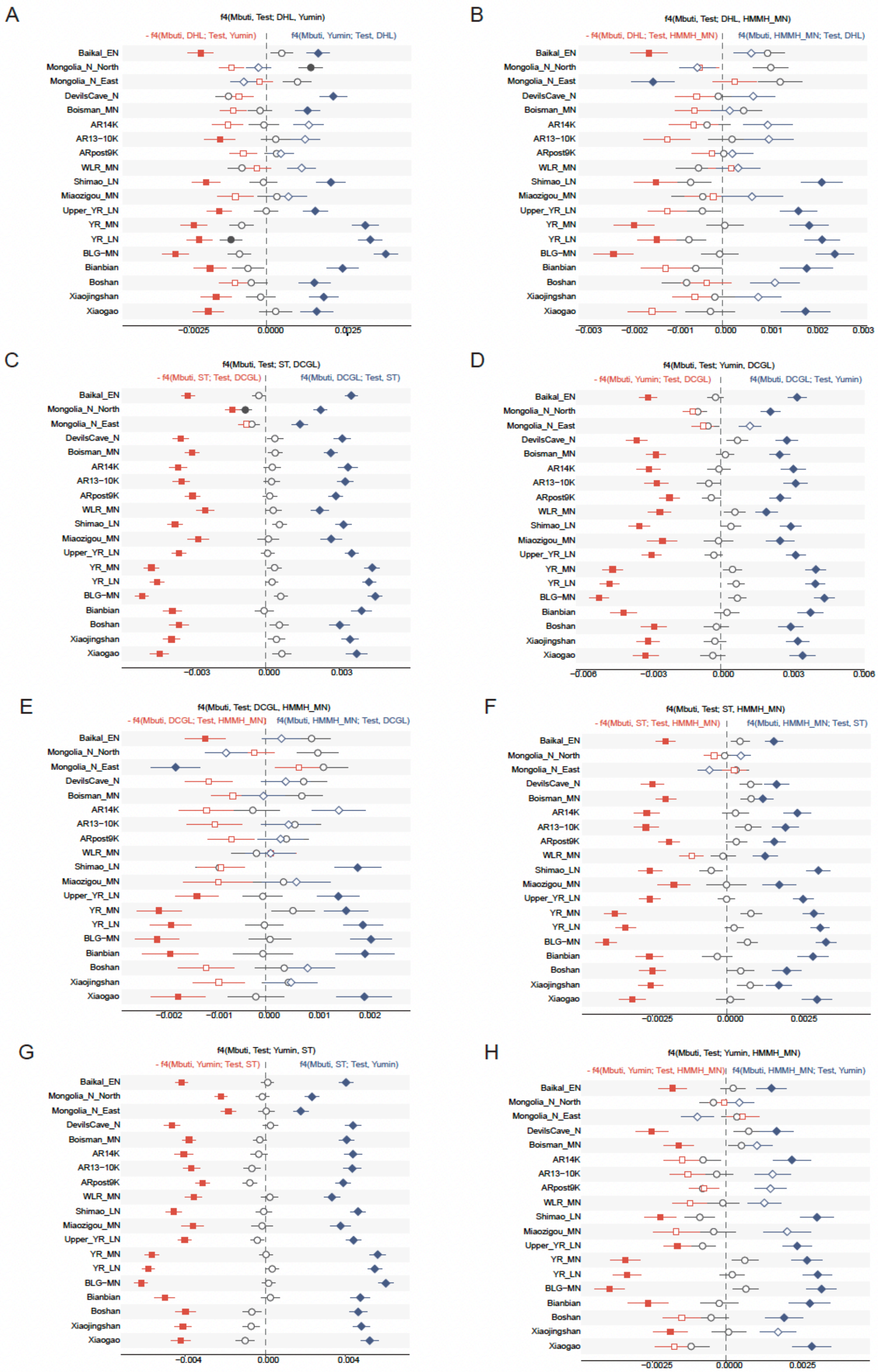

fig. S6. Genetic affinity tests between inland northern East Asian groups. We performed three forms of f4 statistics to examine genetic clustering: *f*4(Mbuti, Pop1; Test, Pop2) > 0, *f*4(Mbuti, Pop2; Test, Pop1) > 0, and *f*4(Mbuti, Test; Pop1, Pop2) ~ 0. The 'Test' label represents populations from Siberia, the Amur River Basin, the West

Liao River Basin, the Yellow River Basin, and Coastal Shandong. The dashed line marks f4 = 0. Solid symbols indicate significant Z-scores ($|Z| > 3$), and error bars denote one standard error. The panels for Pop1 and Pop2 are as follows: (**A**) DHL and Yumin, (**B**) DHL and HMMH_MN, (**C**) ST and DCGL, and (**D**) DCGL and Yumin, (**E**) DCGL and HMMH_MN, (**F**) ST and HMMH_MN, (**G**) ST and Yumin, and (**H**) HMMH_MN and Yumin.

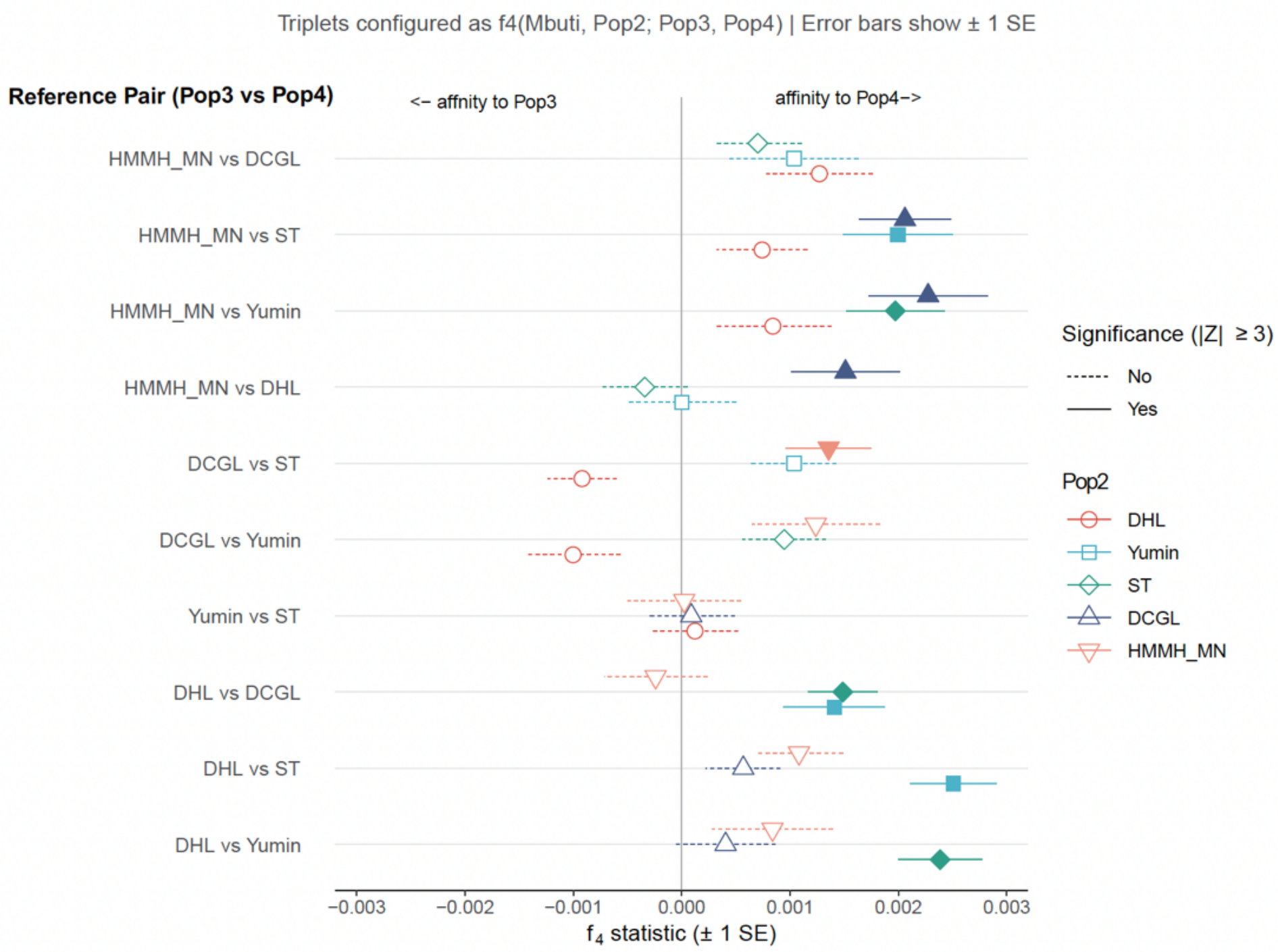

fig. S7. Genetic affinity triplet tests between five key inland northern East Asian groups. We performed three forms of f4 statistics to examine internal relationship: *f*4(Mbuti, group1; group2, group3). The grey line marks f4 = 0. Solid symbols indicate significant Z-scores (|Z| > 3), and error bars denote one standard error.

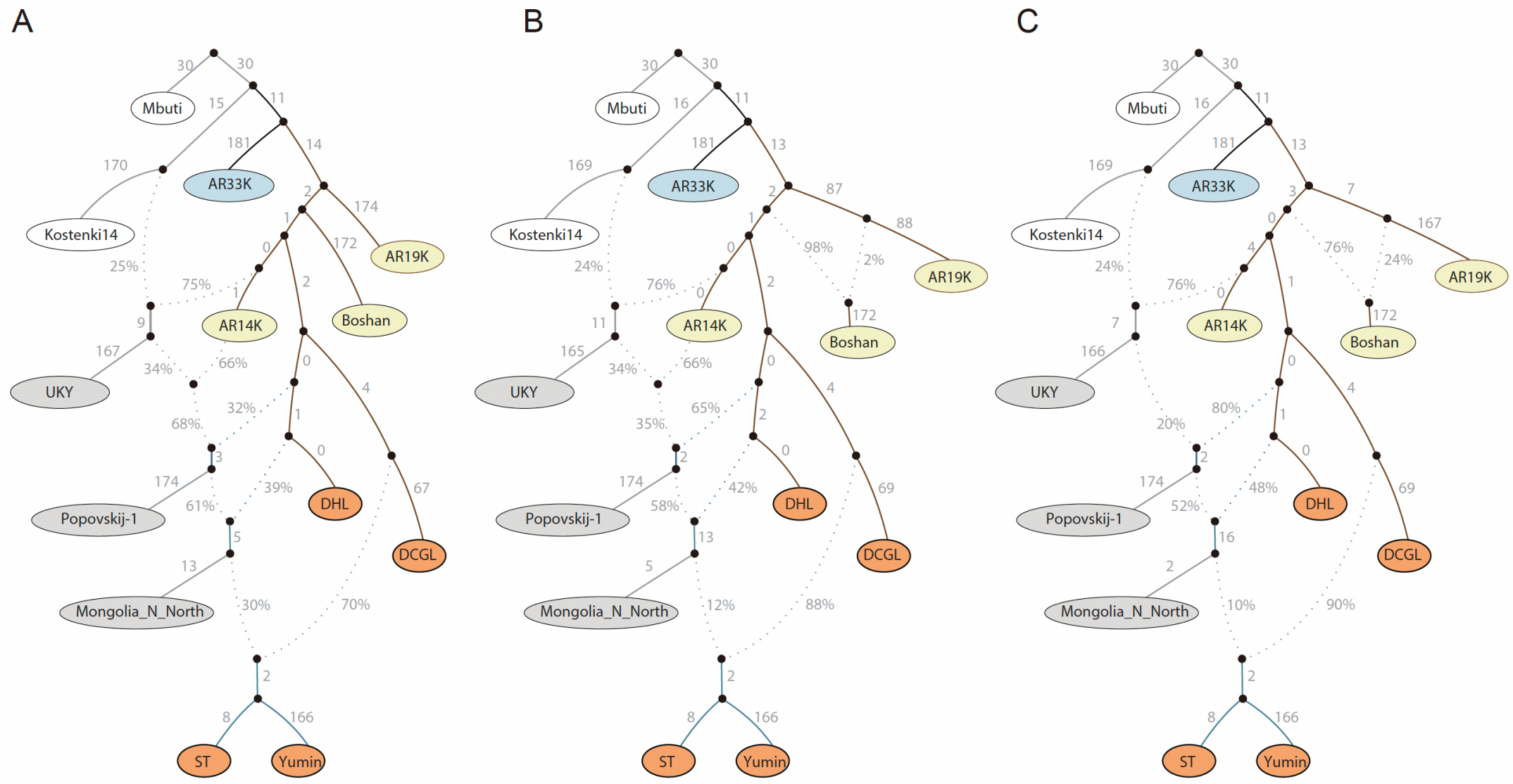

fig. S8. Admixture graph modeling inferred with qpGraph. Dashed lines denote admixture events, and orange colored boxes represent Early-Middle Neolithic millet-farming populations. The worst residual Z-score is 2.77 (**A**), 2.81 (**B**) and 2.75 (**C**).

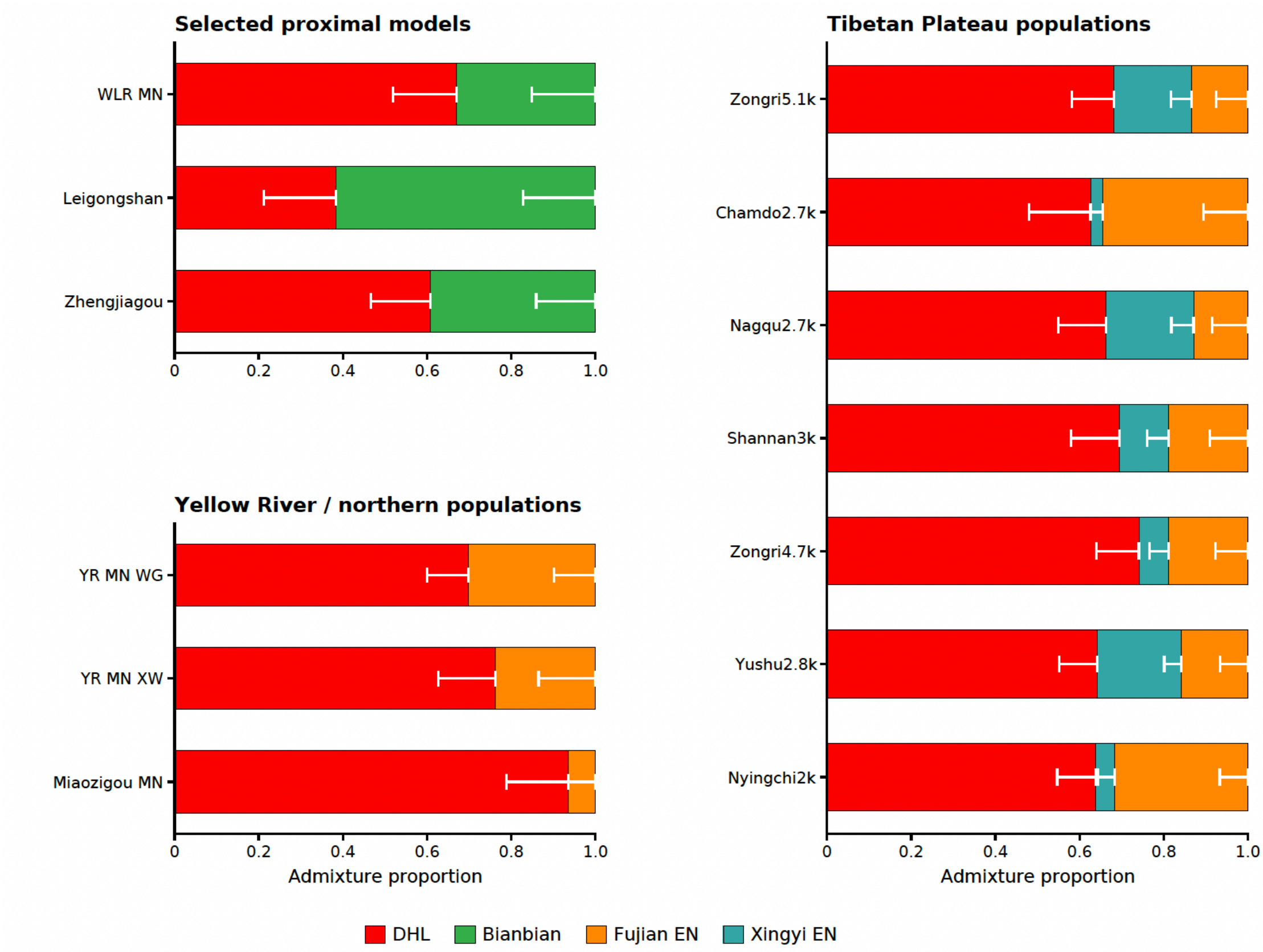

fig. S9. Genetic legacy of DHL-related ancestry in later populations. Stacked bar plots show estimated ancestry proportions inferred from selected feasible qpAdm models. Each horizontal bar represents one target population, with colored segments indicating the proportional contribution of each source population. Error bars denote one standard error for the corresponding ancestry proportion. Models are grouped according to population context. Source populations are indicated by the color legend.

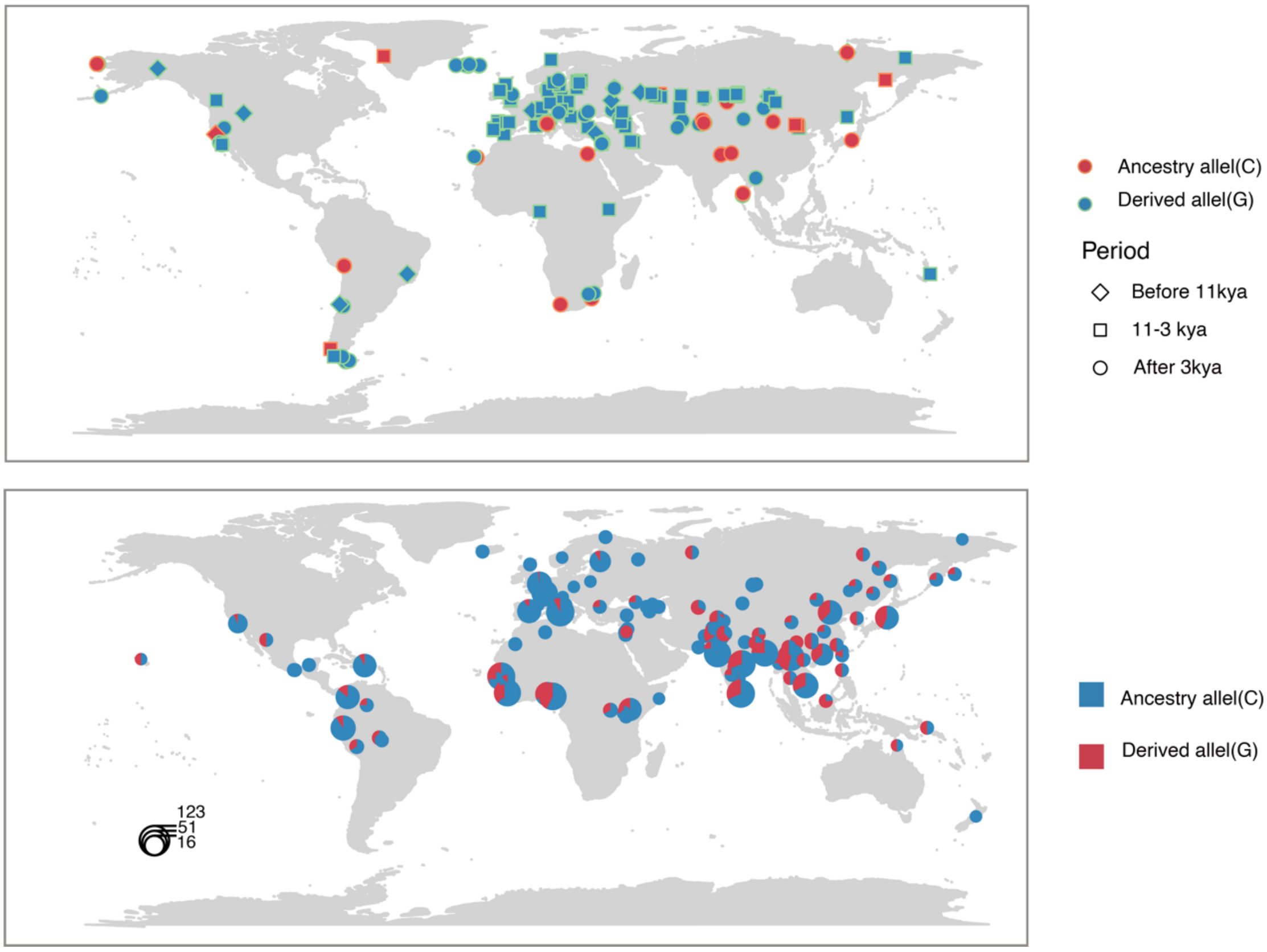

fig. S10. The global distribution of the rs12097901 (EGLN1, C>G) variant. The figure above depicts the distribution of ancient individuals, with each point representing a single individual. Red points indicate ancient individuals possessing the variant. The figure below illustrates the distribution frequency of this variant in present-day populations. Base map outlines were drawn using the world database in the R package maps through map("world").

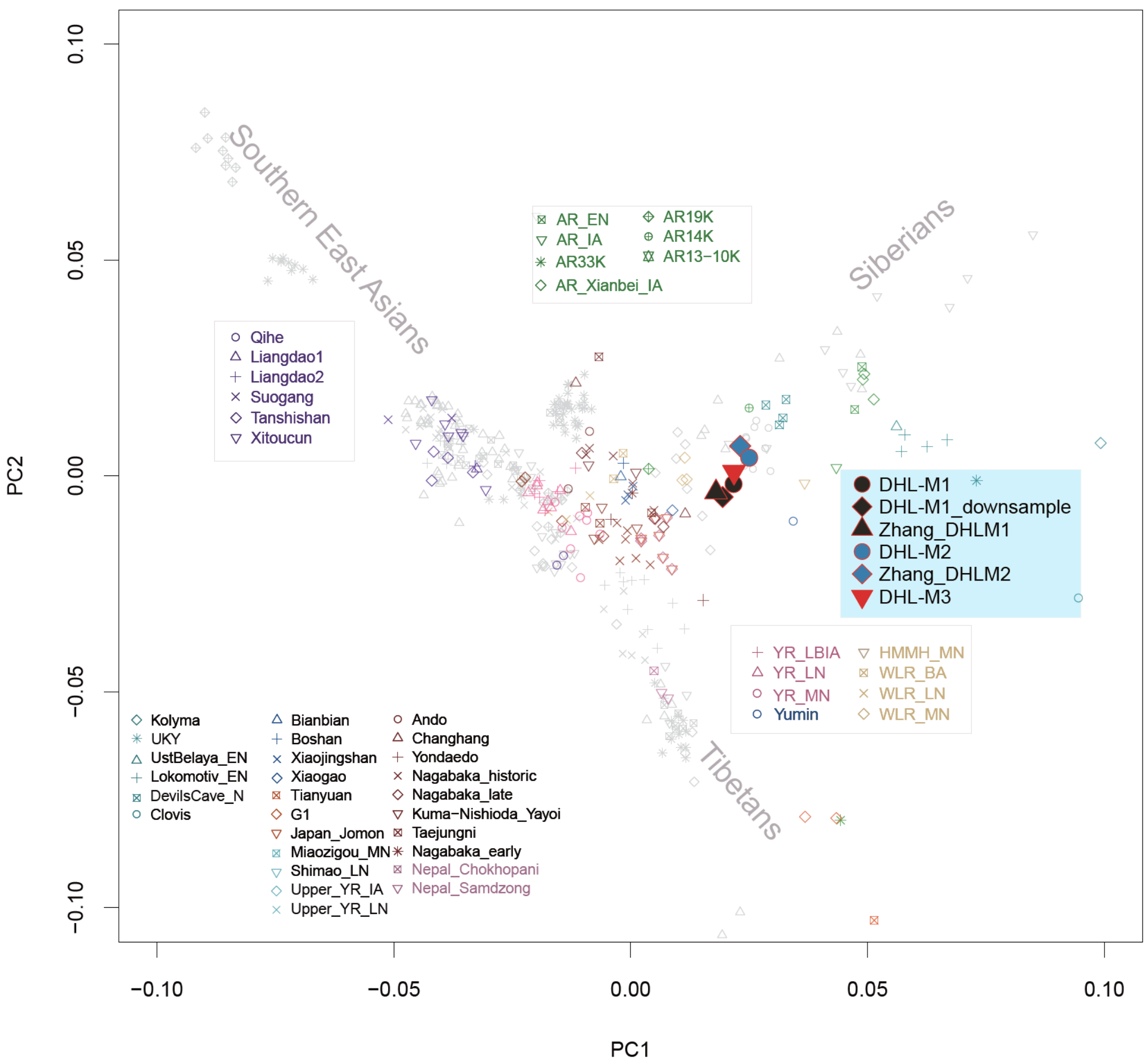

fig. S11. PCA results of the DHL individuals from this study and Zhang et al (*44*). DHL-M1, DHL-M2, and DHL-M3 denote the datasets generated in this study, whereas Zhang_DHLM1 and Zhang_DHLM2 denote the corresponding datasets from Zhang et al (*44*). DHL-M1_downsample represents our DHL-M1 dataset randomly downsampled to approximately match the number of covered 1240K SNPs in Zhang_DHLM1.

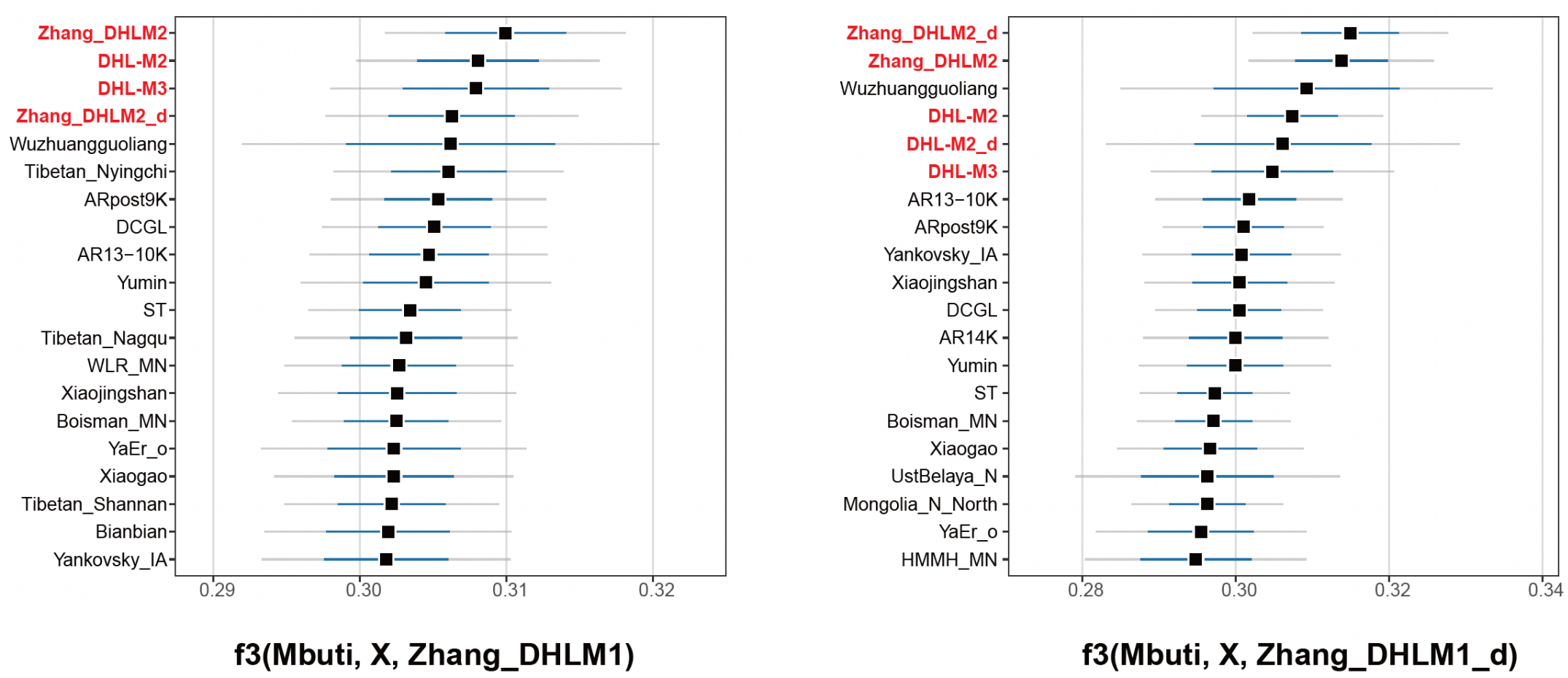

fig. S12. Outgroup-f3 statistics of the form f3(Mbuti; X, Zhang_DHLM1/Zhang_DHLM1_d).

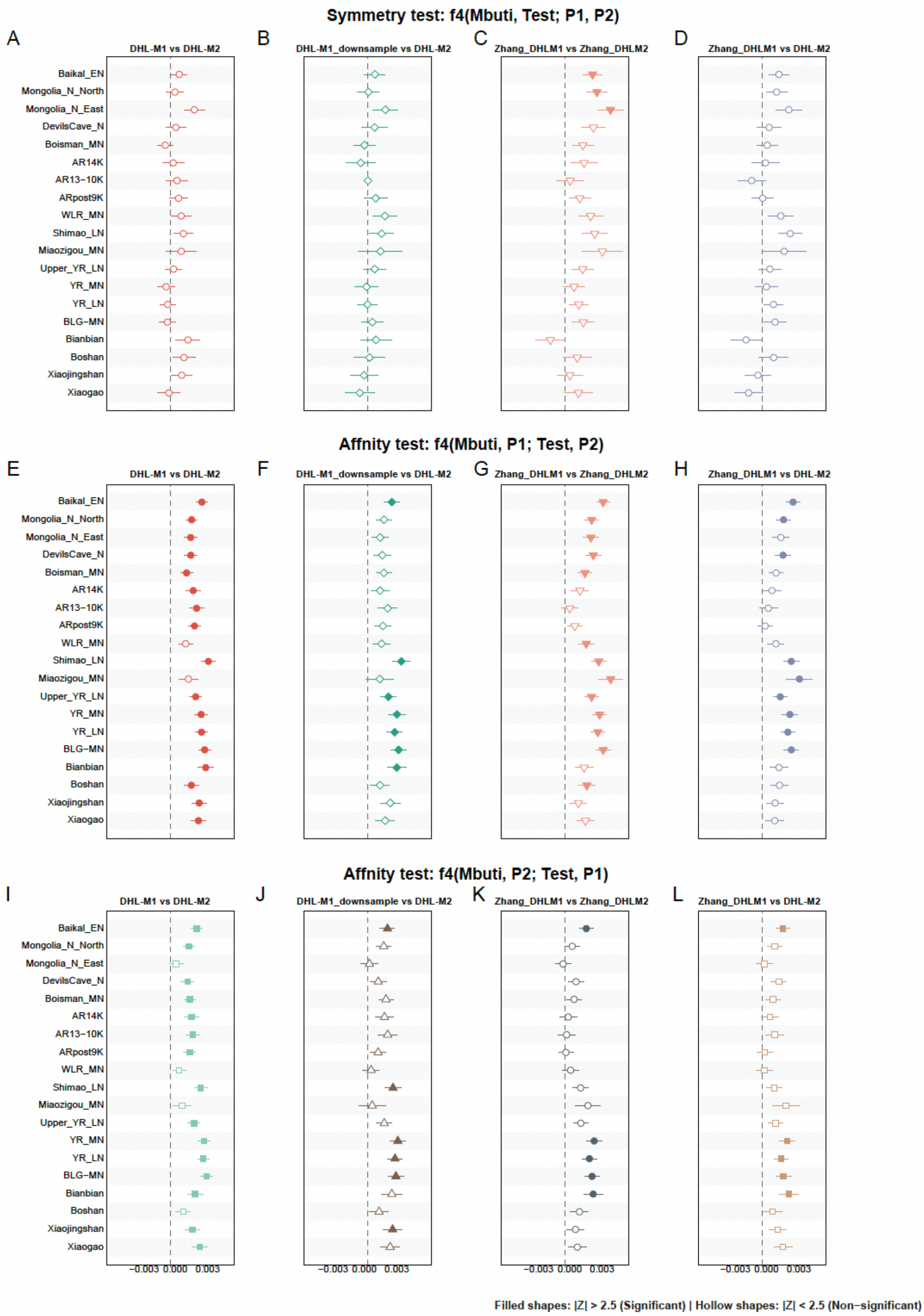

fig. S13. f4-statistic comparisons of the genetic relationships between DHL-M1 and DHL-M2 using datasets from this study and Zhang et al (*44*). (**A–D**) Symmetry tests of the form f4(Mbuti, Test; M1, M2). (**E–H**) Affinity tests of the form f4(Mbuti, M1; Test, M2). (**I–L**) Affinity tests of the form f4(Mbuti, M2; Test, M1).

Table S1. Pairwise mismatch rates (PMR) among the DHL datasets generated in this study and by Zhang et al (*44*).

ID1	ID2	nSNPs	nmismatch	pmismatch
DHL-M1	Zhang_DHLM1	81,600	11,470	0.14056
DHL-M1	Zhang_DHLM1_d	23,380	2,748	0.11754
DHL-M1_d	Zhang_DHLM1	10,081	1,326	0.13153
DHL-M1_d	Zhang_DHLM1_d	3,090	312	0.10097
DHL-M2	Zhang_DHLM2	889,721	102,523	0.11523
DHL-M2_d	Zhang_DHLM2	199,069	22,827	0.11467
DHL-M2	Zhang_DHLM2_d	661,510	75,036	0.11343
DHL-M2_d	Zhang_DHLM2_d	154,162	17,382	0.11275

Supplementary Data files

The following data are provided as a spreadsheet file:
Data S1. Archeological information of the specimens. Including the site, age, isotopic data and skeletal description information of each sample.

Data S2. Summary of lab work and sequence.

Data S3. The sample information for all the ancient individuals sequenced in this study.

Data S4. Dating and isotopic data of some early millet sites shown in Fig. 1F.

Data S5. Z values of f4-statistics, including Data S5.1 to S5.6.

Data S5.1. f4 statistics suggest the genetic cladality among DHL individuals.

Data S5.2. f4 statistics suggest the genetic affinity between DHL and other northern East Asians relative to AR19K.

Data S5.3. f4 statistics suggest inland northern East Asians (DHL, DCGL, ST, Yumin) are similar related to populations from Amur River Basin, Yellow River Basin and costal Shandong, as f4(Mbuti, DHL/DCGL/ST/Yumin/HMMH_MN; nEAs, nEAs) ~ 0.

Data S5.4. f4 statistics suggest the genetic cladality of inland northern East Asian populations (DHL, DCGL, ST, Yumin, HMMN_MN), inluding comparisons with external test populations, alongside internal triplet tests f4(Mbuti, group1; group2, group3)

Data S5.5. f4-statistics suggest the genetic flow between inland northern East Asians (DHL) and Mongolian and Baikal region.

Data S5.6. f4 statistics suggest genetic affinity between DHL and WLR_MN, neighboring millet-farming population.

Data S6. qpAdm modelling for early millet farmers.

Data S7. qpAdm modelling for Baikal/Mongolia hunter-gatherer groups.

Data S8. qpAdm modeling of ancestry in ancient descent populations.

Data S9. Genotypes associated with phenotypes of interest.

Data S10. Phenotype prediction. (**A**).Phenotype prediction with Hirisplex-S. (**B**).Per-SNP results for the 26 lead skin-colour-associated variants (*36*). (**C**). Sample-level summary of the pigmentation polygenic scores analysis and comparison with HIrisPlex-S skin-colour outputs. (**D**). Summary statistics of pigmentation polygenic scores across present-day East Asian populations in the 1000 Genomes Project. (**E**). Individual pigmentation polygenic scores for present-day East Asian individuals from the 1000 Genomes Project.

Data S11. A list of groups used for the population genetic analyses in this study.

REFERENCES

1. Z. Zhao, New archaeobotanic data for the study of the origins of agriculture in China. *Curr. Anthropol.* **52**, S295–S306 (2011).

2. C. J. Stevens, D. Q. Fuller, The spread of agriculture in eastern Asia. *Lang. Dyn. Change* **7**, 152–186 (2017).

3. X. Liu, M. K. Jones, Z. Zhao, G. Liu, T. C. O'Connell, The earliest evidence of millet as a staple crop: New light on neolithic foodways in North China. *Am. J. Phys. Anthropol.* **149**, 283–290 (2012).

4. Y. Li, J. Zhang, X. Zhang, H. Zhao, Agriculture, the environment, and social complexity from the early to late yangshao periods (5000–3000 BC): Insights from macro-botanical remains in North-Central China. *Front. Earth Sci.* **9**, 662391 (2021).

5. C. E. Peterson, X. Lu, R. D. Drennan, D. Zhu, Hongshan chiefly communities in Neolithic northeastern China. *Proc. Natl. Acad. Sci. U.S.A.* **107**, 5756–5761 (2010).

6. M. D. Frachetti, R. N. Spengler, G. J. Fritz, A. N. Mar'yashev, Earliest direct evidence for broomcorn millet and wheat in the central Eurasian steppe region. *Antiquity* **84**, 993–1010 (2010).

7. S. M. Valamoti, Millet, the late comer: On the tracks of Panicum miliaceum in prehistoric Greece. *Archaeol. Anthropol. Sci.* **8**, 51–63 (2016).

8. L. Martin, E. Messager, G. Bedianashvili, N. Rusishvili, E. Lebedeva, C. Longford, R. Hovsepyan, L. Bitadze, M. Chkadua, N. Vanishvili, F. Le Mort, K. Kakhiani, M. Abramishvili, G. Gogochuri, B. Murvanidze, G. Giunashvili, V. Licheli, A. Salavert, G. Andre, E. Herrscher, The place of millet in food globalization during Late Prehistory as evidenced by new bioarchaeological data from the Caucasus. *Sci. Rep.* **11**, 13124 (2021).

9. H. Lu, J. Zhang, K. B. Liu, N. Wu, Y. Li, K. Zhou, M. Ye, T. Zhang, H. Zhang, X. Yang, L. Shen, D. Xu, Q. Li, Earliest domestication of common millet (*Panicum miliaceum*) in East Asia extended to 10,000 years ago. *Proc. Natl. Acad. Sci. U.S.A.* **106**, 7367–7372 (2009).

10. X. Yang, Z. Wan, L. Perry, H. Lu, Q. Wang, C. Zhao, J. Li, F. Xie, J. Yu, T. Cui, T. Wang, M. Li, Q. Ge, Early millet use in northern China. *Proc. Natl. Acad. Sci. U.S.A.* **109**, 3726–3730 (2012).

11. L. Barton, S. D. Newsome, F. H. Chen, H. Wang, T. P. Guilderson, R. L. Bettinger, Agricultural origins and the isotopic identity of domestication in northern China. *Proc. Natl. Acad. Sci. U.S.A.* **106**, 5523–5528 (2009).

12. Z. Zhao, C. Zhao, J. Yu, T. Wang, T. Cui, J. Guo, Flotation results and analysis of plant remains from the Donghulin site in Beijing. *Archaeology (Chinese Series)* **7**, 99–106 (2020).

13. Y. Goldsmith, W. S. Broecker, H. Xu, P. J. Polissar, P. B. deMenocal, N. Porat, J. Lan, P. Cheng, W. Zhou, Z. An, Northward extent of East Asian monsoon covaries with intensity on orbital and millennial timescales. *Proc. Natl. Acad. Sci. U.S.A.* **114**, 1817–1821 (2017).

14. J. Dong, C. C. Shen, X. Kong, H. C. Wang, X. Jiang, Reconciliation of hydroclimate sequences from the Chinese Loess Plateau and low-latitude East Asian Summer Monsoon regions over the past 14,500years. *Palaeogeogr. Palaeoclimatol. Palaeoecol.* **435**, 127–135 (2015).

15. G. Shelach-Lavi, M. Teng, Y. Goldsmith, I. Wachtel, C. J. Stevens, O. Marder, X. Wan, X. Wu, D. Tu, R. Shavit, P. Polissar, H. Xu, D. Q. Fuller, Sedentism and plant cultivation in northeast China emerged during affluent conditions. *PLOS ONE* **14**, e0218751 (2019).

16. C. Leipe, T. Long, E. A. Sergusheva, M. Wagner, P. E. Tarasov, Discontinuous spread of millet agriculture in eastern Asia and prehistoric population dynamics. *Sci. Adv.* **5**, eaax6225 (2019).

17. C. C. Wang, H. Y. Yeh, A. N. Popov, H. Q. Zhang, H. Matsumura, K. Sirak, O. Cheronet, A. Kovalev, N. Rohland, A. M. Kim, S. Mallick, R. Bernardos, D. Tumen, J. Zhao, Y. C. Liu, J. Y. Liu, M. Mah, K. Wang, Z. Zhang, N. Adamski, N. Broomandkhoshbacht, K. Callan, F. Candilio, K. S. D. Carlson, B. J. Culleton, L. Eccles, S. Freilich, D. Keating, A. M. Lawson, K. Mandl, M. Michel, J. Oppenheimer, K. T. Özdoğan, K. Stewardson, S. Wen, S. Yan, F. Zalzala, R. Chuang,

C. J. Huang, H. Looh, C. C. Shiung, Y. G. Nikitin, A. V. Tabarev, A. A. Tishkin, S. Lin, Z. Y. Sun, X. M. Wu, T. L. Yang, X. Hu, L. Chen, H. Du, J. Bayarsaikhan, E. Mijiddorj, D. Erdenebaatar, T. O. Iderkhangai, E. Myagmar, H. Kanzawa-Kiriyama, M. Nishino, K. I. Shinoda, O. A. Shubina, J. Guo, W. Cai, Q. Deng, L. Kang, D. Li, D. Li, R. Lin, R. Nini, L. X. Shrestha, L. Wang, G. Wei, H. Xie, M. Yao, G. Zhang, X. He, R. Yang, M. Hu, S. Robbeets, D. J. Schiffels, L. Kennett, H. Jin, J. Li, R. Krause, D. R. Pinhasi, Genomic insights into the formation of human populations in East Asia. *Nature* **591**, 413–419 (2021).

18. H. Wang, M. A. Yang, S. Wangdue, H. Lu, H. Chen, L. Li, G. Dong, T. Tsring, H. Yuan, W. He, M. Ding, X. Wu, S. Li, N. Tashi, T. Yang, F. Yang, Y. Tong, Z. Chen, Y. He, P. Cao, Q. Dai, F. Liu, X. Feng, T. Wang, R. Yang, W. Ping, Z. Zhang, Y. Gao, M. Zhang, X. Wang, C. Zhang, K. Yuan, A. M. S. Ko, M. Aldenderfer, X. Gao, S. Xu, Q. Fu, Human genetic history on the Tibetan Plateau in the past 5100 years. *Sci. Adv.* **9**, eaax6225 (2023).

19. C. C. Liu, D. Witonsky, A. Gosling, J. H. Lee, H. Ringbauer, R. Hagan, N. Patel, R. Stahl, J. Novembre, M. Aldenderfer, C. Warinner, A. Di Rienzo, C. Jeong, Ancient genomes from the Himalayas illuminate the genetic history of Tibetans and their Tibeto-Burman speaking neighbors. *Nat. Commun.* **13**, 1203 (2022).

20. C. Ning, T. Li, K. Wang, F. Zhang, T. Li, X. Wu, S. Gao, Q. Zhang, H. Zhang, M. J. Hudson, G. Dong, S. Wu, Y. Fang, C. Liu, C. Feng, W. Li, T. Han, R. Li, J. Wei, Y. Zhu, Y. Zhou, C. C. Wang, S. Fan, Z. Xiong, Z. Sun, M. Ye, L. Sun, X. Wu, F. Liang, Y. Cao, X. Wei, H. Zhu, H. Zhou, J. Krause, M. Robbeets, C. Jeong, Y. Cui, Ancient genomes from northern China suggest links between subsistence changes and human migration. *Nat. Commun.* **11**, 2700 (2020).

21. T. Yang, J. He, C. Li, C. Ning, T. Zhang, J. Wang, H. Zhang, G. Wang, X. Jia, C. Zhang, S. Yan, X. Zhang, F. Wu, C. Wu, X. Wu, Y. Pang, Y. Huang, Ancient DNA reveals the population interactions and a Neolithic patrilineal community in Northern Yangtze Region. *Nat. Commun.* **16**, 8728 (2025).

22. J. Xiong, Y. Xu, G. Chen, L. Yang, Y. Zhou, Y. Pan, Z. Wang, J. Bai, B. Zhang, G. Dong, J. Pei, X. Yang, L. Chen, N. Kang, Y. Wu, B. Wang, K. Zhu, P. Du, X. Li, H. Wen, X. Ma, T. Bai, W. Gu, Y. Ye, Q. Wu, X. Chang, J. Tan, L. Gao, D. Ge, B. Li, Y. Yang, W. Feng, Y. Yang, P. Sheng,

H. Meng, R. Wang, J. Zheng, X. Jia, L. Jin, C. C. Wang, S. Wen, The genomic history of East Asian Middle Neolithic millet- and rice-agricultural populations. *Cell Genom.* **5**, 100976 (2025).

23. M. A. Yang, X. Fan, B. Sun, C. Chen, J. Lang, Y. C. Ko, C. H. Tsang, H. Chiu, T. Wang, Q. Bao, X. Wu, M. Hajdinjak, A. M. S. Ko, M. Ding, P. Cao, R. Yang, F. Liu, B. Nickel, Q. Dai, X. Feng, L. Zhang, C. Sun, C. Ning, W. Zeng, Y. Zhao, M. Zhang, X. Gao, Y. Cui, D. Reich, M. Stoneking, Q. Fu, Ancient DNA indicates human population shifts and admixture in northern and southern China. *Science* **369**, 282–288 (2020).

24. X. Mao, H. Zhang, S. Qiao, Y. Liu, F. Chang, P. Xie, M. Zhang, T. Wang, M. Li, P. Cao, R. Yang, F. Liu, Q. Dai, X. Feng, W. Ping, C. Lei, J. W. Olsen, E. A. Bennett, Q. Fu, The deep population history of northern East Asia from the Late Pleistocene to the Holocene. *Cell* **184**, 3256–3266.e13 (2021).

25. D. Zhang, B. Sun, F. Li, X. Zhao, Z. Zhao, J. Li, S. Gao, Y. Li, Q. Quan, N. Berdnikova, I. Berdnikov, S. Chen, F. Zhang, C. Li, C. Jeong, Y. Cui, Neolithic genomes reveal long distance interactions in agropastoral border zone of Yan Mountain Region. *Sci. Bull.* **71**, 148–158 (2026).

26. T. Liu, Z. Zhao, M. Guo, E. A. Bennett, P. Cao, L. Zhuang, Q. Dai, W. Zhang, F. Liu, H. Shi, M. Song, T. Wang, F. Bai, J. Ran, W. Ping, G. Zhang, X. Feng, Q. Fu, The genetic history around the southeastern Mongolian Plateau traces Neolithic cultural diffusions in northern East Asia. *Int. J. Hydrog Energ.* **7**, 101186 (2026).

27. D. J. Cohen, The beginnings of agriculture in China: A multiregional view. *Curr. Anthropol.* **52**, S273–S293 (2011).

28. L. Liu, X. Chen, *The Archaeology of China: From the Late Paleolithic to the Early Bronze Age* (Cambridge University Press 2012).

29. Z. Zhao, The prehistoric Donghulin site in Mentougou District, Beijing. *Archaeology (Chinese Series)* **7**, 97–98 (2006).

30. W. Haak, I. Lazaridis, N. Patterson, N. Rohland, S. Mallick, B. Llamas, G. Brandt, S. Nordenfelt, E. Harney, K. Stewardson, Q. Fu, A. Mittnik, E. Bánffy, C. Economou, M. Francken,

S. Friederich, R. G. Pena, F. Hallgren, V. Khartanovich, A. Khokhlov, M. Kunst, P. Kuznetsov, H. Meller, O. Mochalov, V. Moiseyev, N. Nicklisch, S. L. Pichler, R. Risch, M. A. Rojo Guerra, C. Roth, A. Szécsényi-Nagy, J. Wahl, M. Meyer, J. Krause, D. Brown, D. Anthony, A. Cooper, K. W. Alt, D. Reich, Massive migration from the steppe was a source for Indo-European languages in Europe. *Nature* **522**, 207–211 (2015).

31. Q. Fu, M. Hajdinjak, O. T. Moldovan, S. Constantin, S. Mallick, P. Skoglund, N. Patterson, N. Rohland, I. Lazaridis, B. Nickel, B. Viola, K. Prüfer, M. Meyer, J. Kelso, D. Reich, S. Pääbo, An early modern human from Romania with a recent Neanderthal ancestor. *Nature* **524**, 216–219 (2015).

32. Z. Qiu, X. Wu, M. Guo, G. Wang, Flotation results and analysis of plant remains from the 2018–2019 season at the Xinglong site in Kangbao County, Hebei. *Archaeology (Chinese Series)* **1**, 106–120 (2023).

33. M. Sikora, V. V. Pitulko, V. C. Sousa, M. E. Allentoft, L. Vinner, S. Rasmussen, A. Margaryan, P. de Barros Damgaard, C. de la Fuente, G. Renaud, M. A. Yang, Q. Fu, I. Dupanloup, K. Giampoudakis, D. Nogués-Bravo, C. Rahbek, G. Kroonen, M. Peyrot, H. McColl, S. V. Vasilyev, E. Veselovskaya, M. Gerasimova, E. Y. Pavlova, V. G. Chasnyk, P. A. Nikolskiy, A. V. Gromov, V. I. Khartanovich, V. Moiseyev, P. S. Grebenyuk, A. Y. Fedorchenko, A. I. Lebedintsev, S. B. Slobodin, B. A. Malyarchuk, R. Martiniano, M. Meldgaard, L. Arppe, J. U. Palo, T. Sundell, K. Mannermaa, M. Putkonen, V. Alexandersen, C. Primeau, N. Baimukhanov, R. S. Malhi, K. G. Sjögren, K. Kristiansen, A. Wessman, A. Sajantila, M. M. Lahr, R. Durbin, R. Nielsen, D. J. Meltzer, L. Excoffier, E. Willerslev, The population history of northeastern Siberia since the Pleistocene. *Nature* **570**, 182–188 (2019).

34. H. Yu, M. A. Spyrou, M. Karapetian, S. Shnaider, R. Radzevičiūtė, K. Nägele, G. U. Neumann, S. Penske, J. Zech, M. Lucas, P. LeRoux, P. Roberts, G. Pavlenok, A. Buzhilova, C. Posth, C. Jeong, J. Krause, Paleolithic to bronze age siberians reveal connections with first americans and across Eurasia. *Cell* **181**, 1232–1245.e20 (2020).

35. E. C. Heinrich, L. Wu, E. S. Lawrence, A. M. Cole, C. Anza-Ramirez, F. C. Villafuerte, T. S. Simonson, Genetic variants at the EGLN1 locus associated with high-altitude adaptation in

Tibetans are absent or found at low frequency in highland Andeans. *Ann. Hum. Genet.* **83**, 171–176 (2019).

36. B. Kim, D. S. Kim, J. G. Shin, S. Leem, M. Cho, H. Kim, K. N. Gu, J. Y. Seo, S. W. You, A. R. Martin, S. G. Park, Y. Kim, C. Jeong, N. G. Kang, H. H. Won, Mapping and annotating genomic loci to prioritize genes and implicate distinct polygenic adaptations for skin color. *Nat. Commun.* **15**, 4874 (2024).

37. 1000 Genomes Project Consortium, A. Auton, L. D. Brooks, R. M. Durbin, E. P. Garrison, H. M. Kang, J. O. Korbel, J. L. Marchini, S. McCarthy, G. A. McVean, G. R. Abecasis, A global reference for human genetic variation. *Nature* **526**, 68–74 (2015).

38. Y. Hu, S. Wang, F. Luan, C. Wang, M. P. Richards, Stable isotope analysis of humans from Xiaojingshan site: Implications for understanding the origin of millet agriculture in China. *J. Archaeol. Sci.* **35**, 2960–2965 (2008).

39. Z. Zhao, Z. Gong, J. Li, Z. Zhang, The Sitai Neolithic site in Shangyi County, Hebei. *Archaeology (Chinese Series)* **7**, 14–28+2 (2023).

40. G. W. Crawford, X. Chen, F. Luan, J. Wang, People and plant interaction at the Houli Culture Yuezhuang site in Shandong Province, China. *Holocene* **26**, 1594–1604 (2016).

41. S. Bestel, Y. Bao, H. Zhong, X. Chen, L. Liu, Wild plant use and multi-cropping at the early Neolithic Zhuzhai site in the middle Yellow River region, China. *Holocene* **28**, 195–207 (2018).

42. Q. Yang, X. Li, X. Zhou, K. Zhao, N. Sun, Quantitative reconstruction of summer precipitation using a mid-Holocene $\delta^{13}C$ common millet record from Guanzhong Basin, northern China. *Clim. Past* **12**, 2229–2240 (2016).

43. Z. Deng, The origin and early spread of broomcorn and foxtail millets. *Archaeological Research (Chinese Series)* **1**, 172–214 (2022).

44. G. Zhang, C. Zhao, T. Wang, T. Wang, E. A. Bennett, T. Cui, J. Guo, J. Yu, J. Liu, P. Cao, J. Ran, Q. Dai, T. Liu, F. Liu, F. Bai, X. Feng, W. Ping, W. Wang, Q. Fu, Ancient genomes provide

insight into the Paleolithic-to-Neolithic transition in northern East Asia. *Curr. Biol.* **36**, 1399–1409.e7 (2026).

45. M. Feldman, E. Fernández-Domínguez, L. Reynolds, D. Baird, J. Pearson, I. Hershkovitz, H. May, N. Goring-Morris, M. Benz, J. Gresky, R. A. Bianco, A. Fairbairn, G. Mustafaoğlu, P. W. Stockhammer, C. Posth, W. Haak, C. Jeong, J. Krause, Late Pleistocene human genome suggests a local origin for the first farmers of central Anatolia. *Nat. Commun.* **10**, 1218 (2019).

46. I. Lazaridis, D. Nadel, G. Rollefson, D. C. Merrett, N. Rohland, S. Mallick, D. Fernandes, M. Novak, B. Gamarra, K. Sirak, S. Connell, K. Stewardson, E. Harney, Q. Fu, G. Gonzalez-Fortes, E. R. Jones, S. A. Roodenberg, G. Lengyel, F. Bocquentin, B. Gasparian, J. M. Monge, M. Gregg, V. Eshed, A. S. Mizrahi, C. Meiklejohn, F. Gerritsen, L. Bejenaru, M. Blüher, A. Campbell, G. Cavalleri, D. Comas, P. Froguel, E. Gilbert, S. M. Kerr, P. Kovacs, J. Krause, D. McGettigan, M. Merrigan, D. A. Merriwether, S. O'Reilly, M. B. Richards, O. Semino, M. Shamoon-Pour, G. Stefanescu, M. Stumvoll, A. Tönjes, A. Torroni, J. F. Wilson, L. Yengo, N. A. Hovhannisyan, N. Patterson, R. Pinhasi, D. Reich, Genomic insights into the origin of farming in the ancient Near East. *Nature* **536**, 419–424 (2016).

47. W. L. Merrill, R. J. Hard, J. B. Mabry, G. J. Fritz, K. R. Adams, J. R. Roney, A. C. MacWilliams, The diffusion of maize to the southwestern United States and its impact. *Proc. Natl. Acad. Sci. U.S.A.* **106**, 21019–21026 (2009).

48. X. Zhang, X. Ji, C. Li, T. Yang, J. Huang, Y. Zhao, Y. Wu, S. Ma, Y. Pang, Y. Huang, Y. He, B. Su, A Late Pleistocene human genome from Southwest China. *Curr. Biol.* **32**, 3095–3109.e5 (2022).

49. G. Chaplin, N. G. Jablonski, The human environment and the vitamin D compromise: Scotland as a case study in human biocultural adaptation and disease susceptibility. *Hum. Biol.* **85**, 529–552 (2013).

50. T. A. Brown, D. E. Nelson, J. S. Vogel, J. R. Southon, Improved collagen extraction by modified longin method. *Radiocarbon* **30**, 171–177 (1988).

51. R. E. M. Hedges, G. J. Van Klinken, A review of current approaches in the pretreatment of bone for radiocarbon dating by AMS. *Radiocarbon* **34**, 279–291 (1992).

52. C. Bronk Ramsey, Bayesian analysis of radiocarbon dates. *Radiocarbon* **51**, 337–360 (2009).

53. P. J. Reimer, W. E. N. Austin, E. Bard, A. Bayliss, P. G. Blackwell, C. Bronk Ramsey, M. Butzin, H. Cheng, R. L. Edwards, M. Friedrich, P. M. Grootes, T. P. Guilderson, I. Hajdas, T. J. Heaton, A. G. Hogg, K. A. Hughen, B. Kromer, S. W. Manning, R. Muscheler, J. G. Palmer, C. Pearson, J. van der Plicht, R. W. Reimer, D. A. Richards, E. M. Scott, J. R. Southon, C. S. M. Turney, L. Wacker, F. Adolphi, U. Büntgen, M. Capano, S. M. Fahrni, A. Fogtmann-Schulz, R. Friedrich, P. Köhler, S. Kudsk, F. Miyake, J. Olsen, F. Reinig, M. Sakamoto, A. Sookdeo, S. Talamo, The IntCal20 northern hemisphere radiocarbon age calibration curve (0–55 cal kBP). *Radiocarbon* **62**, 725–757 (2020).

54. X. Zhang, J. Wang, Z. Xian, S. Qiu, Research on ancient human dietary structure. *Archaeology (Chinese Series)*, 158–171 (2003).

55. X. Zhang, Carbon-13 and nitrogen-15 analysis and recent advances in the study of ancient human dietary structure. *Archaeology (Chinese Series)*, 50–56 (2006).

56. X. Zhang, G. Liu, M. Wang, P. Lu, Stable carbon and nitrogen isotope analysis of human bones from the Xinglonggou site. *Cultural Relics South. China* **4**, 185–195 (2017).

57. H. Li, R. Durbin, Fast and accurate short read alignment with Burrows–Wheeler transform. *Bioinformatics* **25**, 1754–1760 (2009).

58. P. Danecek, J. K. Bonfield, J. Liddle, J. Marshall, V. Ohan, M. O. Pollard, A. Whitwham, T. Keane, S. A. McCarthy, R. M. Davies, H. Li, Twelve years of SAMtools and BCFtools. *Gigascience* **10**, giab008 (2021).

59. R. M. Andrews, I. Kubacka, P. F. Chinnery, R. N. Lightowlers, D. M. Turnbull, N. Howell, Reanalysis and revision of the Cambridge Reference Sequence for human mitochondrial DNA. *Nat. Genet.* **23**, 147–147 (1999).

60. G. Renaud, V. Slon, A. T. Duggan, J. Kelso, Schmutzi: Estimation of contamination and endogenous mitochondrial consensus calling for ancient DNA. *Genome Biol.* **16**, 224 (2015).

61. H. Jónsson, A. Ginolhac, M. Schubert, P. L. F. Johnson, L. Orlando, mapDamage2.0: Fast approximate Bayesian estimates of ancient DNA damage parameters. *Bioinformatics* **29**, 1682–1684 (2013).

62. M. Rasmussen, S. L. Anzick, M. R. Waters, P. Skoglund, M. DeGiorgio, T. W. Stafford, S. Rasmussen, I. Moltke, A. Albrechtsen, S. M. Doyle, G. D. Poznik, V. Gudmundsdottir, R. Yadav, A. S. Malaspinas, S. S. W. V, M. E. Allentoft, O. E. Cornejo, K. Tambets, A. Eriksson, P. D. Heintzman, M. Karmin, T. S. Korneliussen, D. J. Meltzer, T. L. Pierre, J. Stenderup, L. Saag, V. M. Warmuth, M. C. Lopes, R. S. Malhi, S. Brunak, T. Sicheritz-Ponten, I. Barnes, M. Collins, L. Orlando, F. Balloux, A. Manica, R. Gupta, M. Metspalu, C. D. Bustamante, M. Jakobsson, R. Nielsen, E. Willerslev, The genome of a Late Pleistocene human from a Clovis burial site in western Montana. *Nature* **506**, 225–229 (2014).

63. M. Rasmussen, M. Sikora, A. Albrechtsen, T. S. Korneliussen, J. V. Moreno-Mayar, G. D. Poznik, C. P. E. Zollikofer, M. S. Ponce de León, M. E. Allentoft, I. Moltke, H. Jónsson, C. Valdiosera, R. S. Malhi, L. Orlando, C. D. Bustamante, T. W. Stafford, D. J. Meltzer, R. Nielsen, E. Willerslev, The ancestry and affiliations of Kennewick Man. *Nature* **523**, 455–458 (2015).

64. M. Lipson, O. Cheronet, S. Mallick, N. Rohland, M. Oxenham, M. Pietrusewsky, T. O. Pryce, A. Willis, H. Matsumura, H. Buckley, K. Domett, G. H. Nguyen, H. H. Trinh, A. A. Kyaw, T. T. Win, B. Pradier, N. Broomandkhoshbacht, F. Candilio, P. Changmai, D. Fernandes, M. Ferry, B. Gamarra, E. Harney, J. Kampuansai, W. Kutanan, M. Michel, M. Novak, J. Oppenheimer, K. Sirak, K. Stewardson, Z. Zhang, P. Flegontov, R. Pinhasi, D. Reich, Ancient genomes document multiple waves of migration in Southeast Asian prehistory. *Science* **361**, 92–95 (2018).

65. M. A. Yang, X. Gao, C. Theunert, H. Tong, A. Aximu-Petri, B. Nickel, M. Slatkin, M. Meyer, S. Pääbo, J. Kelso, Q. Fu, 40,000-year-old individual from asia provides insight into early population structure in Eurasia. *Curr. Biol.* **27**, 3202–3208.e9 (2017).

66. H. McColl, F. Racimo, L. Vinner, F. Demeter, T. Gakuhari, J. V. Moreno-Mayar, G. van Driem, U. G. Wilken, A. Seguin-Orlando, C. de la Fuente Castro, S. Wasef, R. Shoocongdej, V. Souksavatdy, T. Sayavongkhamdy, M. M. Saidin, M. E. Allentoft, T. Sato, A. S. Malaspinas, F. A. Aghakhanian, T. Korneliussen, A. Prohaska, A. Margaryan, P. de Barros Damgaard, S. Kaewsutthi, P. Lertrit, T. M. H. Nguyen, H. C. Hung, T. M. Tran, H. N. Truong, G. H. Nguyen, S. Shahidan, K. Wiradnyana, H. Matsumae, N. Shigehara, M. Yoneda, H. Ishida, T. Masuyama, Y. Yamada, A. Tajima, H. Shibata, A. Toyoda, T. Hanihara, S. Nakagome, T. Deviese, A. M. Bacon, P. Duringer, J. L. Ponche, L. Shackelford, E. Patole-Edoumba, A. T. Nguyen, B. Bellina-Pryce, J. C. Galipaud, R. Kinaston, H. Buckley, C. Pottier, S. Rasmussen, T. Higham, R. A. Foley, M. M. Lahr, L. Orlando, M. Sikora, M. E. Phipps, H. Oota, C. Higham, D. M. Lambert, E. Willerslev, The prehistoric peopling of Southeast Asia. *Science* **361**, 88–92 (2018).

67. C. Jeong, A. T. Ozga, D. B. Witonsky, H. Malmström, H. Edlund, C. A. Hofman, R. W. Hagan, M. Jakobsson, C. M. Lewis, M. S. Aldenderfer, A. Di Rienzo, C. Warinner, Long-term genetic stability and a high-altitude East Asian origin for the peoples of the high valleys of the Himalayan arc. *Proc. Natl. Acad. Sci. U.S.A.* **113**, 7485–7490 (2016).

68. V. Siska, E. R. Jones, S. Jeon, Y. Bhak, H. M. Kim, Y. S. Cho, H. Kim, K. Lee, E. Veselovskaya, T. Balueva, M. Gallego-Llorente, M. Hofreiter, D. G. Bradley, A. Eriksson, R. Pinhasi, J. Bhak, A. Manica, Genome-wide data from two early Neolithic East Asian individuals dating to 7700 years ago. *Sci. Adv.* **3**, e1601877 (2017).

69. P. Skoglund, C. Posth, K. Sirak, M. Spriggs, F. Valentin, S. Bedford, G. R. Clark, C. Reepmeyer, F. Petchey, D. Fernandes, Q. Fu, E. Harney, M. Lipson, S. Mallick, M. Novak, N. Rohland, K. Stewardson, S. Abdullah, M. P. Cox, F. R. Friedlaender, J. S. Friedlaender, T. Kivisild, G. Koki, P. Kusuma, D. A. Merriwether, F. X. Ricaut, J. T. S. Wee, N. Patterson, J. Krause, R. Pinhasi, D. Reich, Genomic insights into the peopling of the Southwest Pacific. *Nature* **538**, 510–513 (2016).

70. P. D. B. Damgaard, N. Marchi, S. Rasmussen, M. Peyrot, G. Renaud, T. Korneliussen, J. V. Moreno-Mayar, M. W. Pedersen, A. Goldberg, E. Usmanova, N. Baimukhanov, V. Loman, L. Hedeager, A. G. Pedersen, K. Nielsen, G. Afanasiev, K. Akmatov, A. Aldashev, A. Alpaslan, G.

Baimbetov, V. I. Bazaliiskii, A. Beisenov, B. Boldbaatar, B. Boldgiv, C. Dorzhu, S. Ellingvag, D. Erdenebaatar, R. Dajani, E. Dmitriev, V. Evdokimov, K. M. Frei, A. Gromov, A. Goryachev, H. Hakonarson, T. Hegay, Z. Khachatryan, R. Khaskhanov, E. Kitov, A. Kolbina, T. Kubatbek, A. Kukushkin, I. Kukushkin, N. Lau, A. Margaryan, I. Merkyte, I. V. Mertz, V. K. Mertz, E. Mijiddorj, V. Moiyesev, G. Mukhtarova, B. Nurmukhanbetov, Z. Orozbekova, I. Panyushkina, K. Pieta, V. Smrčka, I. Shevnina, A. Logvin, K. G. Sjögren, T. Štolcová, A. M. Taravella, K. Tashbaeva, A. Tkachev, T. Tulegenov, D. Voyakin, L. Yepiskoposyan, S. Undrakhbold, V. Varfolomeev, A. Weber, M. A. Wilson Sayres, N. Kradin, M. E. Allentoft, L. Orlando, R. Nielsen, M. Sikora, E. Heyer, K. Kristiansen, E. Willerslev, 137 ancient human genomes from across the Eurasian steppes. *Nature* **557**, 369–374 (2018).

71. P. Flegontov, N. E. Altınışık, P. Changmai, N. Rohland, S. Mallick, N. Adamski, D. A. Bolnick, N. Broomandkhoshbacht, F. Candilio, B. J. Culleton, O. Flegontova, T. M. Friesen, C. Jeong, T. K. Harper, D. Keating, D. J. Kennett, A. M. Kim, T. C. Lamnidis, A. M. Lawson, I. Olalde, J. Oppenheimer, B. A. Potter, J. Raff, R. A. Sattler, P. Skoglund, K. Stewardson, E. J. Vajda, S. Vasilyev, E. Veselovskaya, M. G. Hayes, D. H. O'Rourke, J. Krause, R. Pinhasi, D. Reich, S. Schiffels, Palaeo-Eskimo genetic ancestry and the peopling of Chukotka and North America. *Nature* **570**, 236–240 (2019).

72. V. M. Narasimhan, N. Patterson, P. Moorjani, N. Rohland, R. Bernardos, S. Mallick, I. Lazaridis, N. Nakatsuka, I. Olalde, M. Lipson, A. M. Kim, L. M. Olivieri, A. Coppa, M. Vidale, J. Mallory, V. Moiseyev, E. Kitov, J. Monge, N. Adamski, N. Alex, N. Broomandkhoshbacht, F. Candilio, K. Callan, O. Cheronet, B. J. Culleton, M. Ferry, D. Fernandes, S. Freilich, B. Gamarra, D. Gaudio, M. Hajdinjak, É. Harney, T. K. Harper, D. Keating, A. M. Lawson, M. Mah, K. Mandl, M. Michel, M. Novak, J. Oppenheimer, N. Rai, K. Sirak, V. Slon, K. Stewardson, F. Zalzala, Z. Zhang, G. Akhatov, A. N. Bagashev, A. Bagnera, B. Baitanayev, J. Bendezu-Sarmiento, A. A. Bissembaev, G. L. Bonora, T. T. Chargynov, T. Chikisheva, P. K. Dashkovskiy, A. Derevianko, M. Dobeš, K. Douka, N. Dubova, M. N. Duisengali, D. Enshin, A. Epimakhov, A. V. Fribus, D. Fuller, A. Goryachev, A. Gromov, S. P. Grushin, B. Hanks, M. Judd, E. Kazizov, A. Khokhlov, A. P. Krygin, E. Kupriyanova, P. Kuznetsov, D. Luiselli, F. Maksudov, A. M. Mamedov, T. B. Mamirov, C. Meiklejohn, D. C. Merrett, R. Micheli, O. Mochalov, S. Mustafokulov, A. Nayak, D. Pettener, R. Potts, D. Razhev, M. Rykun, S. Sarno, T. M.

Savenkova, K. Sikhymbaeva, S. M. Slepchenko, O. A. Soltobaev, N. Stepanova, S. Svyatko, K. Tabaldiev, M. Teschler-Nicola, A. A. Tishkin, V. V. Tkachev, S. Vasilyev, P. Velemínský, D. Voyakin, A. Yermolayeva, M. Zahir, V. S. Zubkov, A. Zubova, V. S. Shinde, C. Lalueza-Fox, M. Meyer, D. Anthony, N. Boivin, K. Thangaraj, D. J. Kennett, M. Frachetti, R. Pinhasi, D. Reich, The formation of human populations in South and central Asia. *Science* **365**, eaat7487 (2019).

73. V. Shinde, V. M. Narasimhan, N. Rohland, S. Mallick, M. Mah, M. Lipson, N. Nakatsuka, N. Adamski, N. Broomandkhoshbacht, M. Ferry, A. M. Lawson, M. Michel, J. Oppenheimer, K. Stewardson, N. Jadhav, Y. J. Kim, M. Chatterjee, A. Munshi, A. Panyam, P. Waghmare, Y. Yadav, H. Patel, A. Kaushik, K. Thangaraj, M. Meyer, N. Patterson, N. Rai, D. Reich, An ancient harappan genome lacks ancestry from steppe pastoralists or iranian farmers. *Cell* **179**, 729–735.e10 (2019).

74. Q. Fu, C. Posth, M. Hajdinjak, M. Petr, S. Mallick, D. Fernandes, A. Furtwängler, W. Haak, M. Meyer, A. Mittnik, B. Nickel, A. Peltzer, N. Rohland, V. Slon, S. Talamo, I. Lazaridis, M. Lipson, I. Mathieson, S. Schiffels, P. Skoglund, A. P. Derevianko, N. Drozdov, V. Slavinsky, A. Tsybankov, R. G. Cremonesi, F. Mallegni, B. Gély, E. Vacca, M. R. G. Morales, L. G. Straus, C. Neugebauer-Maresch, M. Teschler-Nicola, S. Constantin, O. T. Moldovan, S. Benazzi, M. Peresani, D. Coppola, M. Lari, S. Ricci, A. Ronchitelli, F. Valentin, C. Thevenet, K. Wehrberger, D. Grigorescu, H. Rougier, I. Crevecoeur, D. Flas, P. Semal, M. A. Mannino, C. Cupillard, H. Bocherens, N. J. Conard, K. Harvati, V. Moiseyev, D. G. Drucker, J. Svoboda, M. P. Richards, D. Caramelli, R. Pinhasi, J. Kelso, N. Patterson, J. Krause, S. Pääbo, D. Reich, The genetic history of ice age Europe. *Nature* **534**, 200–205 (2016).

75. M. E. Allentoft, M. Sikora, K. G. Sjögren, S. Rasmussen, M. Rasmussen, J. Stenderup, P. B. Damgaard, H. Schroeder, T. Ahlström, L. Vinner, A. S. Malaspinas, A. Margaryan, T. Higham, D. Chivall, N. Lynnerup, L. Harvig, J. Baron, P. D. Casa, P. Dąbrowski, P. R. Duffy, A. V. Ebel, A. Epimakhov, K. Frei, M. Furmanek, T. Gralak, A. Gromov, S. Gronkiewicz, G. Grupe, T. Hajdu, R. Jarysz, V. Khartanovich, A. Khokhlov, V. Kiss, J. Kolář, A. Kriiska, I. Lasak, C. Longhi, G. McGlynn, A. Merkevicius, I. Merkyte, M. Metspalu, R. Mkrtchyan, V. Moiseyev, L. Paja, G. Pálfi, D. Pokutta, Ł. Pospieszny, T. D. Price, L. Saag, M. Sablin, N. Shishlina, V. Smrčka, V. I. Soenov, V. Szeverényi, G. Tóth, S. V. Trifanova, L. Varul, M. Vicze, L. Yepiskoposyan, V.

Zhitenev, L. Orlando, T. Sicheritz-Pontén, S. Brunak, R. Nielsen, K. Kristiansen, E. Willerslev, Population genomics of Bronze Age Eurasia. *Nature* **522**, 167–172 (2015).

76. M. Sikora, A. Seguin-Orlando, V. C. Sousa, A. Albrechtsen, T. Korneliussen, A. Ko, S. Rasmussen, I. Dupanloup, P. R. Nigst, M. D. Bosch, G. Renaud, M. E. Allentoft, A. Margaryan, S. V. Vasilyev, E. V. Veselovskaya, S. B. Borutskaya, T. Deviese, D. Comeskey, T. Higham, A. Manica, R. Foley, D. J. Meltzer, R. Nielsen, L. Excoffier, M. Mirazon Lahr, L. Orlando, E. Willerslev, Ancient genomes show social and reproductive behavior of early Upper Paleolithic foragers. *Science* **358**, 659–662 (2017).

77. P. Flegontov, P. Changmai, A. Zidkova, M. D. Logacheva, N. E. Altınışık, O. Flegontova, M. S. Gelfand, E. S. Gerasimov, E. E. Khrameeva, O. P. Konovalova, T. Neretina, Y. V. Nikolsky, G. Starostin, V. V. Stepanova, I. V. Travinsky, M. Tříska, P. Tříska, T. V. Tatarinova, Genomic study of the Ket: A Paleo-Eskimo-related ethnic group with significant ancient North Eurasian ancestry. *Sci. Rep.* **6**, 20768 (2016).

78. T. Wang, W. Wang, G. Xie, Z. Li, X. Fan, Q. Yang, X. Wu, P. Cao, Y. Liu, R. Yang, F. Liu, Q. Dai, X. Feng, X. Wu, L. Qin, F. Li, W. Ping, L. Zhang, M. Zhang, Y. Liu, X. Chen, D. Zhang, Z. Zhou, Y. Wu, H. Shafiey, X. Gao, D. Curnoe, X. Mao, E. A. Bennett, X. Ji, M. A. Yang, Q. Fu, Human population history at the crossroads of East and Southeast Asia since 11,000 years ago. *Cell* **184**, 3829–3841.e21 (2021).

79. M. Robbeets, R. Bouckaert, M. Conte, A. Savelyev, T. Li, D. I. An, K. I. Shinoda, Y. Cui, T. Kawashima, G. Kim, J. Uchiyama, J. Dolińska, S. Oskolskaya, K. Y. Yamano, N. Seguchi, H. Tomita, H. Takamiya, H. Kanzawa-Kiriyama, H. Oota, H. Ishida, R. Kimura, T. Sato, J. H. Kim, B. Deng, R. Bjørn, S. Rhee, K. D. Ahn, I. Gruntov, O. Mazo, J. R. Bentley, R. Fernandes, P. Roberts, I. R. Bausch, L. Gilaizeau, M. Yoneda, M. Kugai, R. A. Bianco, F. Zhang, M. Himmel, M. J. Hudson, C. Ning, Triangulation supports agricultural spread of the Transeurasian languages. *Nature* **599**, 616–621 (2021).

80. K. Shinoda, H. Kanzawa-Kiriyama, T. Kakuda, N. Adachi, Mitochondrial DNA analysis of human skeletal remains from the Shimomotoyama site. *Anthropological Science (Japanese Series)* **127**, 65–72 (2019).

81. K. Shinoda, T. Kakuda, N. Adachi, Genetic characteristics of the Yayoi people based on ancient DNA analysis. *Bull. Natl. Mus. Jpn. Hist.* **219**, 163–177 (2020).

82. N. P. Cooke, V. Mattiangeli, L. M. Cassidy, K. Okazaki, C. A. Stokes, S. Onbe, S. Hatakeyama, K. Machida, K. Kasai, N. Tomioka, A. Matsumoto, M. Ito, Y. Kojima, D. G. Bradley, T. Gakuhari, S. Nakagome, Ancient genomics reveals tripartite origins of Japanese populations. *Sci. Adv.* **7**, eabh2419 (2021).

83. F. Broushaki, M. G. Thomas, V. Link, S. López, L. van Dorp, K. Kirsanow, Z. Hofmanová, Y. Diekmann, L. M. Cassidy, D. Díez-del-Molino, A. Kousathanas, C. Sell, H. K. Robson, R. Martiniano, J. Blöcher, A. Scheu, S. Kreutzer, R. Bollongino, D. Bobo, H. Davoudi, O. Munoz, M. Currat, K. Abdi, F. Biglari, O. E. Craig, D. G. Bradley, S. Shennan, K. R. Veeramah, M. Mashkour, D. Wegmann, G. Hellenthal, J. Burger, Early neolithic genomes from the eastern fertile crescent. *Science* **353**, 499–503 (2016).

84. I. Mathieson, I. Lazaridis, N. Rohland, S. Mallick, N. Patterson, S. A. Roodenberg, E. Harney, K. Stewardson, D. Fernandes, M. Novak, K. Sirak, C. Gamba, E. R. Jones, B. Llamas, S. Dryomov, J. Pickrell, J. L. Arsuaga, J. M. B. de Castro, E. Carbonell, F. Gerritsen, A. Khokhlov, P. Kuznetsov, M. Lozano, H. Meller, O. Mochalov, V. Moiseyev, M. A. R. Guerra, J. Roodenberg, J. M. Vergès, J. Krause, A. Cooper, K. W. Alt, D. Brown, D. Anthony, C. Lalueza-Fox, W. Haak, R. Pinhasi, D. Reich, Genome-wide patterns of selection in 230 ancient Eurasians. *Nature* **528**, 499–503 (2015).

85. M. Raghavan, P. Skoglund, K. E. Graf, M. Metspalu, A. Albrechtsen, I. Moltke, S. Rasmussen, T. W. Stafford Jr., L. Orlando, E. Metspalu, M. Karmin, K. Tambets, S. Rootsi, R. Mägi, P. F. Campos, E. Balanovska, O. Balanovsky, E. Khusnutdinova, S. Litvinov, L. P. Osipova, S. A. Fedorova, M. I. Voevoda, M. DeGiorgio, T. Sicheritz-Ponten, S. Brunak, S. Demeshchenko, T. Kivisild, R. Villems, R. Nielsen, M. Jakobsson, E. Willerslev, Upper palaeolithic siberian genome reveals dual ancestry of Native Americans. *Nature* **505**, 87–91 (2014).

86. D. Massilani, L. Skov, M. Hajdinjak, B. Gunchinsuren, D. Tseveendorj, S. Yi, J. Lee, S. Nagel, B. Nickel, T. Devièse, T. Higham, M. Meyer, J. Kelso, B. M. Peter, S. Pääbo, Denisovan ancestry and population history of early East Asians. *Science* **370**, 579–583 (2020).

87. S. Mallick, H. Li, M. Lipson, I. Mathieson, M. Gymrek, F. Racimo, M. Zhao, N. Chennagiri, S. Nordenfelt, A. Tandon, P. Skoglund, I. Lazaridis, S. Sankararaman, Q. Fu, N. Rohland, G. Renaud, Y. Erlich, T. Willems, C. Gallo, J. P. Spence, Y. S. Song, G. Poletti, F. Balloux, G. van Driem, P. de Knijff, I. G. Romero, A. R. Jha, D. M. Behar, C. M. Bravi, C. Capelli, T. Hervig, A. Moreno-Estrada, O. L. Posukh, E. Balanovska, O. Balanovsky, S. Karachanak-Yankova, H. Sahakyan, D. Toncheva, L. Yepiskoposyan, C. Tyler-Smith, Y. Xue, M. S. Abdullah, A. Ruiz-Linares, C. M. Beall, A. Di Rienzo, C. Jeong, E. B. Starikovskaya, E. Metspalu, J. Parik, R. Villems, B. M. Henn, U. Hodoglugil, R. Mahley, A. Sajantila, G. Stamatoyannopoulos, J. T. S. Wee, R. Khusainova, E. Khusnutdinova, S. Litvinov, G. Ayodo, D. Comas, M. F. Hammer, T. Kivisild, W. Klitz, C. A. Winkler, D. Labuda, M. Bamshad, L. B. Jorde, S. A. Tishkoff, W. S. Watkins, M. Metspalu, S. Dryomov, R. Sukernik, L. Singh, K. Thangaraj, S. Pääbo, J. Kelso, N. Patterson, D. Reich, The Simons Genome Diversity Project: 300 genomes from 142 diverse populations. *Nature* **538**, 201–206 (2016).

88. D. Lu, H. Lou, K. Yuan, X. Wang, Y. Wang, C. Zhang, Y. Lu, X. Yang, L. Deng, Y. Zhou, Q. Feng, Y. Hu, Q. Ding, Y. Yang, S. Li, L. Jin, Y. Guan, B. Su, L. Kang, S. Xu, Ancestral origins and genetic history of tibetan highlanders. *Am. J. Hum. Genet.* **99**, 580–594 (2016).

89. A. Seguin-Orlando, T. S. Korneliussen, M. Sikora, A. S. Malaspinas, A. Manica, I. Moltke, A. Albrechtsen, A. Ko, A. Margaryan, V. Moiseyev, T. Goebel, M. Westaway, D. Lambert, V. Khartanovich, J. D. Wall, P. R. Nigst, R. A. Foley, M. M. Lahr, R. Nielsen, L. Orlando, E. Willerslev, Genomic structure in Europeans dating back at least 36,200 years. *Science* **346**, 1113–1118 (2014).

90. Q. Fu, M. Meyer, X. Gao, U. Stenzel, H. A. Burbano, J. Kelso, S. Pääbo, DNA analysis of an early modern human from Tianyuan Cave, China. *Proc. Natl. Acad. Sci. U.S.A.* **110**, 2223–2227 (2013).

91. T. S. Korneliussen, A. Albrechtsen, R. Nielsen, ANGSD: Analysis of next generation sequencing data. *BMC Bioinformatics* **15**, 356 (2014).

92. A. Dür, N. Huber, W. Parson, Fine-tuning phylogenetic alignment and haplogrouping of mtDNA sequences. *Int. J. Mol. Sci.* **22**, 5747 (2021).

93. N. Patterson, A. L. Price, D. Reich, Population structure and eigenanalysis. *PLOS Genet.* **2**, e190 (2006).

94. E. Y. Durand, N. Patterson, D. Reich, M. Slatkin, Testing for ancient admixture between closely related populations. *Mol. Biol. Evol.* **28**, 2239–2252 (2011).

95. N. Patterson, P. Moorjani, Y. Luo, S. Mallick, N. Rohland, Y. Zhan, T. Genschoreck, T. Webster, D. Reich, Ancient admixture in human history. *Genetics* **192**, 1065–1093 (2012).

96. C. J. E. Ingram, M. F. Elamin, C. A. Mulcare, M. E. Weale, A. Tarekegn, T. O. Raga, E. Bekele, F. M. Elamin, M. G. Thomas, N. Bradman, D. M. Swallow, A novel polymorphism associated with lactose tolerance in Africa: Multiple causes for lactase persistence? *Hum. Genet.* **120**, 779–788 (2007).

97. S. A. Tishkoff, F. A. Reed, A. Ranciaro, B. F. Voight, C. C. Babbitt, J. S. Silverman, K. Powell, H. M. Mortensen, J. B. Hirbo, M. Osman, M. Ibrahim, S. A. Omar, G. Lema, T. B. Nyambo, J. Ghori, S. Bumpstead, J. K. Pritchard, G. A. Wray, P. Deloukas, Convergent adaptation of human lactase persistence in Africa and Europe. *Nat. Genet.* **39**, 31–40 (2007).

98. H. Li, S. Gu, X. Cai, W. C. Speed, A. J. Pakstis, E. I. Golub, J. R. Kidd, K. K. Kidd, Ethnic related selection for an ADH class I variant within East Asia. *PLOS ONE* **3**, e1881 (2008).

99. I. Olalde, M. E. Allentoft, F. Sánchez-Quinto, G. Santpere, C. W. K. Chiang, M. DeGiorgio, J. Prado-Martinez, J. A. Rodríguez, S. Rasmussen, J. Quilez, O. Ramírez, U. M. Marigorta, M. Fernández-Callejo, M. E. Prada, J. M. V. Encinas, R. Nielsen, M. G. Netea, J. Novembre, R. A. Sturm, P. Sabeti, T. Marquès-Bonet, A. Navarro, E. Willerslev, C. Lalueza-Fox, Derived immune and ancestral pigmentation alleles in a 7,000-year-old Mesolithic European. *Nature* **507**, 225–228 (2014).

100. S. Wilde, A. Timpson, K. Kirsanow, E. Kaiser, M. Kayser, M. Unterländer, N. Hollfelder, I. D. Potekhina, W. Schier, M. G. Thomas, J. Burger, Direct evidence for positive selection of skin, hair, and eye pigmentation in Europeans during the last 5,000 y. *Proc. Natl. Acad. Sci. U.S.A.* **111**, 4832–4837 (2014).

101. H. L. Norton, R. A. Kittles, E. Parra, P. McKeigue, X. Mao, K. Cheng, V. A. Canfield, D. G. Bradley, B. McEvoy, M. D. Shriver, Genetic evidence for the convergent evolution of light skin in Europeans and East Asians. *Mol. Biol. Evol.* **24**, 710–722 (2006).

102. S. Walsh, F. Liu, A. Wollstein, L. Kovatsi, A. Ralf, A. Kosiniak-Kamysz, W. Branicki, M. Kayser, The HIrisPlex system for simultaneous prediction of hair and eye colour from DNA. *Forensic Sci. Int. Genet.* **7**, 98–115 (2013).

103. L. Chaitanya, K. Breslin, S. Zuñiga, L. Wirken, E. Pośpiech, M. Kukla-Bartoszek, T. Sijen, P. D. Knijff, F. Liu, W. Branicki, M. Kayser, S. Walsh, The HIrisPlex-S system for eye, hair and skin colour prediction from DNA: Introduction and forensic developmental validation. *Forensic Sci. Int. Genet.* **35**, 123–135 (2018).

104. T. Chen, X. Chen, S. Zhang, J. Zhu, B. Tang, A. Wang, L. Dong, Z. Zhang, C. Yu, Y. Sun, L. Chi, H. Chen, S. Zhai, Y. Sun, L. Lan, X. Zhang, J. Xiao, Y. Bao, Y. Wang, Z. Zhang, W. Zhao, The genome sequence archive family: Toward explosive data growth and diverse data types. *Genomics Proteomics Bioinformatics* **19**, 578–583 (2021).

105. X. Bai, Y. Bao, S. Bei, C. Bu, R. Cao, Y. Cao, H. Cen, J. Chao, F. Chen, H. Chen, K. Chen, M. Chen, M. Chen, M. Chen, Q. Chen, R. Chen, S. Chen, T. Chen, X. Chen, X. Chen, Y. Cheng, Y. Chu, Q. Cui, L. Dong, Z. Du, G. Duan, S. Fan, Z. Fan, X. Fang, Z. Fang, Z. Feng, S. Fu, F. Gao, G. Gao, H. Gao, W. Gao, X. Gao, X. Gao, X. Gao, J. Gong, J. Gong, Y. Gou, S. Gu, A.-Y. Guo, G. Guo, X. Guo, C. Han, D. Hao, L. Hao, Q. He, S. He, S. He, W. Hu, K. Huang, T. Huang, X. Huang, Y. Huang, P. Jia, Y. Jia, C. Jiang, M. Jiang, S. Jiang, T. Jiang, X. Jiang, E. Jin, W. Jin, H. Kang, H. Kang, D. Kong, L. Lan, W. Lei, C.-Y. Li, C. Li, C. Li, H. Li, J. Li, J. Li, L. Li, P. Li, R. Li, X. Li, Y. Li, Y. Li, Z. Li, X. Liao, S. Lin, Y. Lin, Y. Ling, B. Liu, C.-J. Liu, D. Liu, G.-H. Liu, L. Liu, S. Liu, W. Liu, X. Liu, X. Liu, Y. Liu, Y. Liu, M. Lu, T. Lu, H. Luo, H. Luo, M. Luo, S. Luo, X. T. Luo, L. Ma, Y. Ma, J. Mai, J. Meng, X. Meng, Y. Meng, Y. Meng, W. Miao, Y.-R. Miao, L. Ni, Z. Nie, G. Niu, X. Niu, Y. Niu, R. Pan, S. Pan, D. Peng, J. Peng, J. Qi, Y. Qi, Q. Qian, Y. Qin, H. Qu, J. Ren, J. Ren, Z. Sang, K. Shang, W.-K. Shen, Y. Shen, Y. Shi, S. Song, T. Song, T. Su, J. Sun, Y. Sun, Y. Sun, Y. Sun, B. Tang, D. Tang, Q. Tang, Z. Tang, D. Tian, F. Tian, W. Tian, Z. Tian, A. Wang, G. Wang, G. Wang, J. Wang, J. Wang, P. Wang, P. Wang, W. Wang, Y.

Wang, Y. Wang, Y. Wang, Y. Wang, Z. Wang, H. Wei, Y. Wei, Z. Wei, D. Wu, G. Wu, S. Wu, S. Wu, W. Wu, W. Wu, Z. Wu, Z. Xia, J. Xiao, L. Xiao, Y. Xiao, G. Xie, G.-Y. Xie, J. Xie, Y. Xie, J. Xiong, Z. Xiong, D. Xu, S. Xu, T. Xu, T. Xu, Y. Xue, Y. Xue, C. Yan, D. Yang, F. Yang, F. Yang, H. Yang, J. Yang, K. Yang, N. Yang, Q.-Y. Yang, S. Yang, X. Yang, X. Yang, X. Yang, Y.-G. Yang, W. Ye, C. Yu, F. Yu, S. Yu, C. Yuan, H. Yuan, J. Zeng, S. Zhai, C. Zhang, F. Zhang, G. Zhang, M. Zhang, P. Zhang, Q. Zhang, R. Zhang, S. Zhang, W. Zhang, W. Zhang, W. Zhang, X. Zhang, X. Zhang, Y. Zhang, Y. Zhang, Y. Zhang, Y. E. Zhang, Y. Zhang, Z. Zhang, Z. Zhang, D. Zhao, F. Zhao, G. Zhao, M. Zhao, W. Zhao, W. Zhao, X. Zhao, Y. Zhao, Y. Zhao, Z. Zhao, X. Zheng, Y. Zheng, C. Zhou, H. Zhou, X. Zhou, X. Zhou, Y. Zhou, Y. Zhou, J. Zhu, L. Zhu, R. Zhu, T. Zhu, W. Zong, D. Zou, Z. Zuo, CNCB-NGDC Members and Partners, Database resources of the National Genomics Data Center, China National Center for Bioinformation in 2024. *Nucleic Acids Res.* **52**, D18–D32 (2024).

106. J. Xue, S. Hao, Observation of dental morphological characteristics of Donghulin No. 4. *Acta Anthropol. Sin.* **29**, 253–263 (2010).

107. J. He, C. Zhao, J. Yu, T. Cui, T. Wang, J. Guo, Y. Yuan, X. Yun, Q. Yang, Physical evolution and biocultural adaptation of human skeletons from the Donghulin site in Beijing. *Archaeology (Chinese Series)* **11**, 90–98 (2020).

108. W. Zhang, Preliminary study on mid-Neolithic cultural remains from the southern edge of the Inner Mongolian Plateau. *Northern Cultural Relics (Chinese Series)* **1**, 51–59 (2023).

109. H. Wei, P. Wang, W. Liu, Brief report on the excavation of the Sitai Neolithic site in Shangyi County, Hebei. *Archaeology (Chinese Series)* **4**, 3–15 (2018).

110. S. Peyrégne, B. M. Peter, AuthentiCT: A model of ancient DNA damage to estimate the proportion of present-day DNA contamination. *Genome Biol.* **21**, 246 (2020).
